# Generation of Human iPSC-Derived Astrocytes with a mature star-shaped phenotype for CNS modeling

**DOI:** 10.1007/s12015-022-10376-2

**Published:** 2022-04-30

**Authors:** Dimitrios Voulgaris, Polyxeni Nikolakopoulou, Anna Herland

**Affiliations:** 1 Science for Life Laboratory, Division of Nanobiotechnology, Department of Protein Science., Royal Institute of Technology (KTH), 171 65 Solna, Sweden; 2 AIMES - Center for the Advancement of Integrated Medical and Engineering Sciences (AIMES), Karolinska Institutet and KTH Royal Institute of Technology, Stockholm, Sweden; 3 Department of Neuroscience, Karolinska Institutet, SE-171 77 Stockholm, Sweden

**Keywords:** hiAstrocytes, hiPSCs, Neuroepithelial Stem Cells, EAAT1, NES, Glutathione, ICAM-1

## Abstract

The generation of astrocytes from human induced pluripotent stem cells has been hampered by either prolonged differentiation—spanning over two months—or by shorter protocols that generate immature astrocytes, devoid of salient mature astrocytic traits pivotal for central nervous system (CNS) modeling. We directed stable hiPSC-derived neuroepithelial stem cells to human iPSC-derived Astrocytes (hiAstrocytes) with a high percentage of star-shaped cells by orchestrating an astrocytic-tuned culturing environment in 28 days. We employed RT-qPCR and ICC to validate the astrocytic commitment of the neuroepithelial stem cells. To evaluate the inflammatory phenotype, we challenged the hiAstrocytes with the pro-inflammatory cytokine IL-1β (interleukin 1 beta) and quantitatively assessed the secretion profile of astrocyte-associated cytokines and the expression of intercellular adhesion molecule 1 (ICAM-1). Finally, we quantitatively assessed the capacity of hiAstrocytes to synthesize and export the antioxidant glutathione. In under 28 days, the generated cells express canonical and mature astrocytic markers, denoted by the expression of GFAP, AQP4 and ALDH1L1. In addition, the notion of a mature phenotype is reinforced by the expression of both astrocytic glutamate transporters EAAT1 and EAAT2. Thus, hiAstrocytes have a mature phenotype that encompasses traits critical in CNS modeling, including glutathione synthesis and secretion, upregulation of ICAM-1 and a cytokine secretion profile on a par with human fetal astrocytes. This protocol generates a multifaceted astrocytic model suitable for in vitro CNS disease modeling and personalized medicine.

**Graphical abstract:**

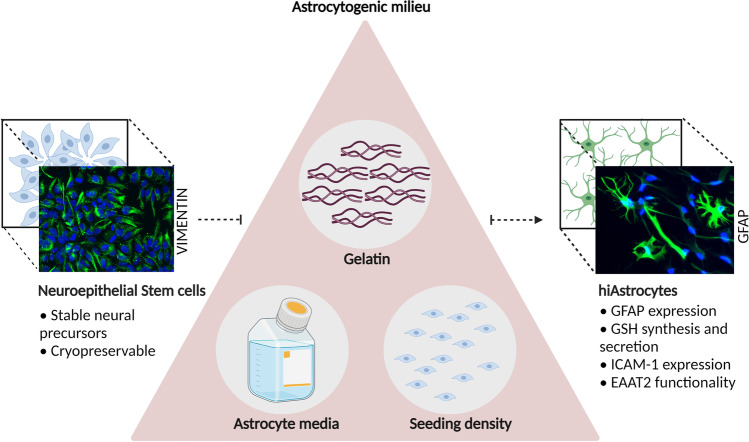

**Supplementary Information:**

The online version contains supplementary material available at 10.1007/s12015-022-10376-2.

## Introduction

Astrocytes have long been considered as merely cellular scaffolds—i.e., the brain’s glue. However, recent studies point out that astrocytes have a prominent role in health and disease; once considered a recycler of neurotransmitters and a supply cabinet for neurons, this star-shaped cell type assumes an ever-growing role in health and disease [1–3].

Astrocytes are a truly versatile brain cell type, regulating a plethora of cellular processes such as glutamate clearance, provision of antioxidants ,e.g. glutathione (GSH) to neurons and relay inflammatory signals. Additionally, astrocytes actively regulate synaptic transmission [1, 4], and they are involved in the pathophysiology of numerous neurodegenerative diseases [3].

Importantly, mouse and human astrocytes are transcriptionally and functionally different [5]. Human astrocytes are larger and have more elaborate processes than mouse astrocytes [6]. Mouse and human inflammatory responses to insults such as trauma and burn share minimal correlation [7] and, more specifically, in the CNS, mouse and human astrocytes are differentially activated by inflammatory stimuli [8] and respond very differently to oxidative stress [9]. Therapeutic interventions in mouse models inadvertently fail to translate into potential treatments in humans [10, 11]. Consequently, over 90% of CNS drug candidates in animal trials fail to successfully display the same therapeutic effects in human clinical trials [12].

The need to model interactions of the CNS necessitates reliable and sustainable (i.e., non-primary) sources of human astrocytes. The extent of information extracted from an in vitro study is limited by the model used; hence, a holistic view of astrocytes in pathology demands the concurrent existence of a spectrum of traits. In amyotrophic lateral sclerosis (ALS), the EAAT2 is lost in astrocytes [13], while astrocytes undergo morphological changes that extend to their processes in various pathological conditions [14–17]. Upon inflammatory stimuli, astrocytes not only secrete cytokines but also upregulate adhesion proteins such as ICAM-1 [18]. The provision of GSH is also altered in many pathological conditions [19–23].

To recapitulate pathological conditions in vitro, an astrocytic model, among others, should: 1) exhibit mature inflammatory responses, 2) synthesize and secrete GSH, 3) express and have functionally active EAAT1 and EAAT2 glutamate transporters and 4) have a star-like morphology. These traits are critical for astrocyte pathophysiology in brain diseases.

Stem cell technology enabled numerous differentiation protocols that generate human iPSC-derived astrocytes, bypassing the need for primary sources and bringing forth human models that can potentially capture in higher biofidelity human astrocytes than mouse models. Currently, astrocyte generation is hampered by either extensive differentiations (> 60 days) [24–27] or by shortened differentiation protocols (< 60 days) that lack a star-shaped morphology, GSH synthesis/secretion and a direct inflammatory comparison to primary astrocytes [28, 29]. More importantly, none of the reported short astrocytic protocols encompass a mature profile (in terms of GSH, glutamate functionality and ICAM-1 expression) on a par with primary astrocytes, the astrocytic source that shares the greatest in vivo proximity. There are, however, short protocols with genome editing approaches, such as the work of Neyrinck et al. [30]. Nevertheless, these approaches require extensive resources.

The use of neural precursors as a starting stage enables robust and reproducible protocols while contributing to shorter differentiation times for the generation of astrocytes. TCW et al. brought forward a compelling notion ﻿based on the motto” we are what we eat” they applied a primary astrocytic media on neural progenitors to induce astrocytic phenotype [31, 32]. However, that protocol is dependent on differentiating transient, unstable neural precursor entities, as exemplified by their limited self-renewal (up to 16 passages), a common trait of neural progenitors generated through dual-SMAD inhibition [33]. The dependence on early passages that are a transient stage could hamper reproducibility, scalability, and wide applicability of the astrocyte protocols.

This transient neural stem cell state can become stable neuroepithelial stem cells (NES) after p.20 [34]. NES are truly stable (up to at least 100 passages) [35] neural progenitors and could pose an ideal starting point for astrocyte differentiation. Our recent efforts in astrocyte generation from NES yielded GFAP-negative cells that lack EAAT2 functionality and IL-6 secretion [36, 37]. Therefore, we envisioned that we could generate mature astrocytes with in vivo-like morphology and functionality by providing an astrocytic-tuned milieu to NES. An astrocytogenic milieu comprises a suitable differentiation media, an astrocytic-tuned extracellular matrix (ECM) coating and appropriate cell-to-cell contact[38].

Here, we report on a differentiation strategy that unleashes the astrocytic potential of stable iPS-derived neuroepithelial stem cells. By day 28 and without any sorting steps, hiAstrocytes feature a star-shaped morphology, display inflammatory potency and active uptake of both astrocytic glutamate transporters (EAAT1 and EAAT2), and an mRNA and protein profile that resembles human astrocytes. Additionally, we demonstrate that hiAstrocytes synthesize and secrete glutathione. This astrocytic model can be used for CNS disease modeling since it encompasses a multitude of traits that are altered during pathological conditions.

## Materials and Methods

All reagents were purchased from Thermo Fisher Scientific, MA, USA unless otherwise stated. Reagents and catalog numbers are listed in Supplementary Table 1.

All processes were done according to the manufacturer’s instructions unless otherwise stated. For all processes, DPBS refers to DPBS with Ca^++^ and Mg^++^ unless otherwise stated.

### Cell Culture

#### Human Neuroepithelial Stem Cells

Neuroepithelial stem cells (NES) lines Control 9 [39], Control 7 [40], and AF22 [35] (detailed information for each line can be found in Supplementary Table 2) were provided by the iPS Core Facility (Karolinska Institute). All lines were cultured and passaged until p.23–24 in DMEM: F12 Glutamax supplemented with N2 1:100, B27 1:1000, 10 ng/ml bFGF (R&D Systems, MN, USA) and 10 ng/ml EGF (Sigma Aldrich, MO, USA) (the complete media is termed N2B27) on double-coated poly-L-ornithine (PLO) (20 ug/ml Sigma Aldrich, MO, USA) and murine Engelbreth-Holm-Swarm sarcoma derived laminin (L2020, 1:500, Sigma Aldrich, MO, USA) flasks. Culture vessels were coated overnight with PLO, washed thoroughly × 2 with DPBS (w/o Ca^++^ and Mg^++^) and then coated overnight with L2020; all coating dilutions were done in DPBS (w/o Ca^++^ and Mg^++^). After p.23–24, NES were cultured in N2B27 with bFGF (10 ng/ml) and low EGF (1 ng/ml). NES culturing was done in flasks (T12.5—T75), and N2B27 media with growth factors was used within one week.

NES were passaged at 1:4–1:5, every 3–4 days. Briefly, cells were washed with DPBS (w/o Ca^++^ and Mg^++^) and then incubated with trypLE for 3–4 min. Next, TrypLE was deactivated using equal volumes of defined trypsin inhibitor (DTI) and DMEM: F12 glutamax (1:1:1), spun down at 200 g for 4 min and resuspended in N2B27.

The day after passaging, bFGF (10 ng/ml) and EGF (10 ng/ml for passages up to p.# 23–24 and 1 ng/ml for the passages after p.# 23–24) were spiked in the cultures, and the day after the media was completely replenished. This motif of cell feeding was done throughout the NES culture.

#### Astrocyte Differentiation

**Fig. 1:**
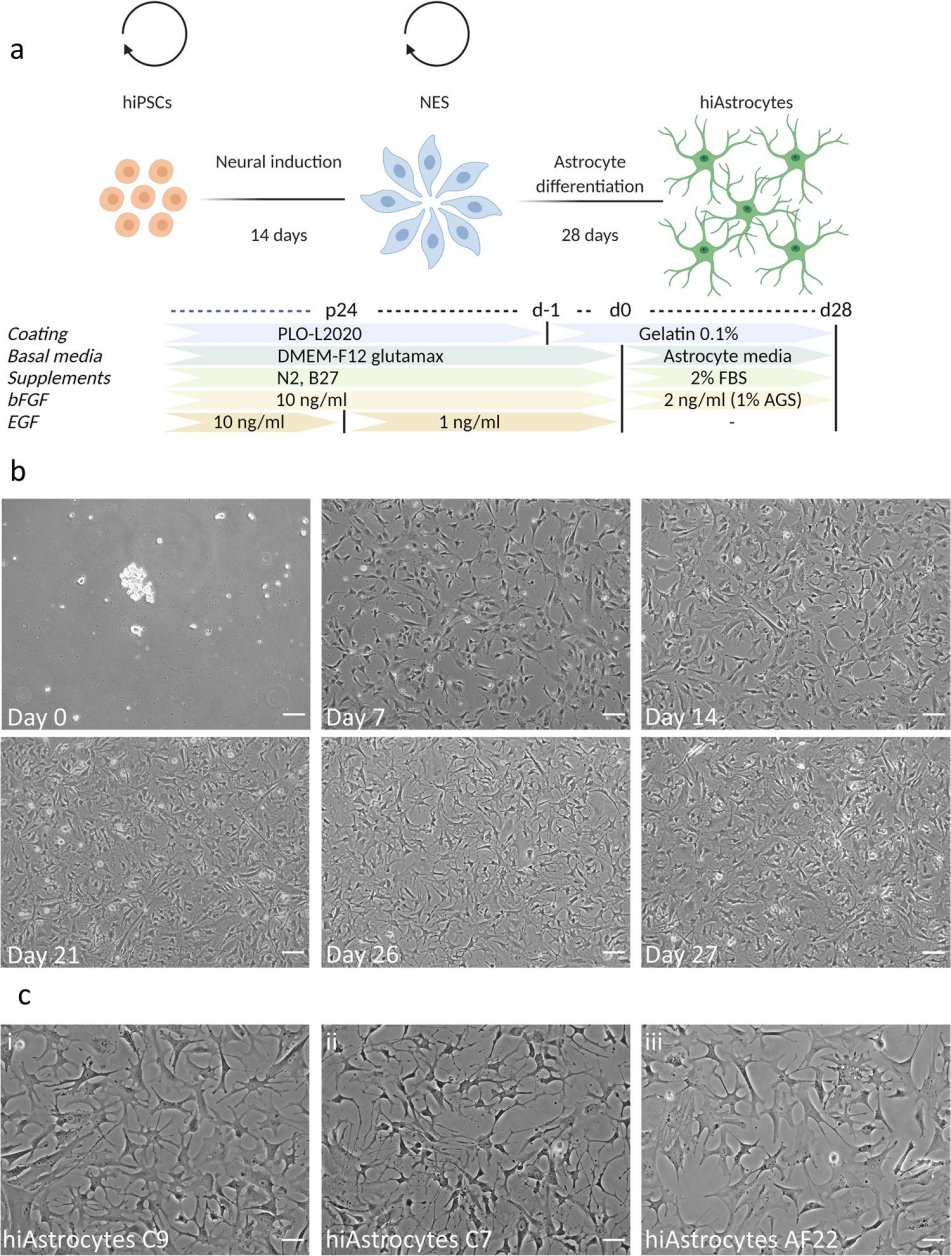
Neuroepithelial stem cells generate star-shaped astrocytes in 28 days under astrocytogenic conditions. a) Schematic presentation of the duration of the protocol used to derive neuroepithelial stem cells and hiAstrocytes. b) Brightfield images of NES C9 for differentiation days 0,7,14, 21, 26 and 27. Scale bar 100 μm. c) Brightfield images of hiAstrocytes C9 (i) hiAstrocytes C7 (ii) and hiAstrocytes AF22 (iii) on day 26 of differentiation. Scale bar 50 μm

On day -2, 6-well plates were coated with 0.1% (w/v) gelatin (Thermo Fisher or ScienCell) at 37 °C. On day-1, NES were passaged using trypLE for 4 min, and trypLE was deactivated by adding DTI at 1:4 (trypLE:DTI). Cells were spun down at 200 g for 3 min and resuspended in N2B27 with bFGF (10 ng/ml) and low EGF (1 ng/ml). NES were seeded at 30 K/cm^2^ in the gelatin-coated 6-well plates. The day after (Fig. 1b, day 0, NES C9), cells formed small aggregates or were loosely attaching. Media was carefully removed, and cells were washed once with DPBS before adding AM media supplemented with 1% AGS and 2% FBS. Media changes were done every other day, and cells were passaged 1–2 days upon reaching 95% confluency, except for the first passage, where cells were passaged on day 6. For all passages, cells were consistently seeded out at 30 K/cm^2^ except when stated otherwise.

All differentiations were carried out using NES with p.# > 30, except for the data presented in Supplementary Fig. 2d, where NES were p.#13. For all assays in well plates, plates were double coated with PDL (50 ug/ml) overnight, washed once with water and then coated at 37 °C with gelatin overnight.

Specifically for Supplementary Fig. 1a-f, astrocyte differentiation was done according to TCW et al. (2017). Briefly, on day -1, NES were seeded at 15 K/cm^2^ in N2B27 (10 ng/ml bFGF and 1 ng/ml EGF) in Matrigel-coated 6-well plates. The day after (day 0), the medium was changed to AM medium with 1% AGS and 2% FBS. Cells were passaged at 15 K/cm^2^ in Matrigel-coated plates once they reached 90% confluency. After 28 days, the cells were collected for qPCR analysis.

#### Spontaneously Differentiated Cells

For spontaneously differentiated cells (SDCs), NES (p.# > 30) were detached using trypLE for 4 min, spun down at 200 g for 4 min, and were seeded out at 30 K/cm^2^ in double-coated PLO (20 ug/ml) and L2020 (1:100) in N2B27 (low EGF). The day after, the media was changed to DMEM: F12 glutamax with N2 1:100 and B27 1:100. After that, the media was completely changed every other day. Cells were split once during the differentiation (day 7) at 45 K/cm^2^ on PLO-L2020 plates. After the first passage, the media was changed every 3 to 4 days. DMEM: F12 glutamax with N2 1:100 and B27 1:100 was first warmed at 37 °C, 10 ng/ml BDNF and 10 ng/ml GDNF (both R&D Systems, MN, USA) were added to the media before complete media change. On day 28, cells were harvested.

#### Human Fetal Astrocytes

Human fetal astrocytes (HFA) were cultured in ScienCell Media AM supplemented with 2% FBS, 1% AGS and 1% P/S. HFAs until passage 7 were used for experiments. Cells were passaged once they reached confluency with a 1:4 ratio. Briefly, cells were washed with DPBS (w/o Ca^++^ and Mg^++^) and then incubated with trypLE for 3–4 min. Next, trypLE was deactivated using DTI 1:1 (TrypLE:DTI) spun down at 200 g for 4 min and resuspended in complete AM media.

### Immunocytochemistry

Cells were initially washed with DPBS and fixed with 4% PFA (VWR, PA, USA) for 10 min at room temperature. After washing twice with DPBS, cells were incubated with blocking buffer (10% goat serum and 0.1% Triton X-100 in DPBS) for an hour. Primary antibody incubation was done in dilution buffer (10% blocking buffer in DPBS) overnight at 4 °C. Secondary antibody incubation was done in dilution buffer at room temperature for an hour. After 2 × washes with DPBS, cells were stained with Hoechst (1:2000) in dilution buffer. Cells were washed 3 × and imaged with ImageXpress Micro (Molecular Devices, CA, USA). Quantification of astrocytic markers and astrocytic processes was done by the Multi-Wavelength Cell Scoring and Neurite Outgrowth module, respectively. A detailed list of the antibodies used can be found in Supplementary Table 3.

### mRNA Expression Analysis

Cells were collected, lysed and total RNA was extracted using the RNeasy Mini kit (Qiagen, Germany); RNA was quantified using MySpec (VWR, PA, USA), and cDNA synthesis was carried out using the High-capacity RNA-to-cDNA kit on a thermal cycler (VWR, PA, USA). TaqMan probes of interest were incubated with cDNA samples in Fast Advanced Master Mix. For all samples, GAPDH TaqMan probes were included as housekeeping gene reference. Samples were run on a BioRad CFX96 Touch Real-Time PCR Detection System using the multiplex option for superior Ct quantification. Depending on the graph presented, either ΔCt or ΔΔCts were determined. Samples that lacked Ct values (e.g., *RELN*, *GFAP* for all NES lines) or had values over 35 were assigned a Ct value of 35 for the ΔΔCt analysis to be possible (or to minimize overestimation of results). PCA was done with R using the prcomp function (R Core Team (2021). R: A language and environment for statistical computing. R Foundation for Statistical Computing, Vienna, Austria. URL https://www.R-project.org/) and hierarchical clustering with the Morpheus software using average linkage clustering, person correlation (Morpheus, https://software.broadinstitute.org/morpheus). A list of the TaqMan probes used can be found in Supplementary Table 4.

### Glutamate Uptake

NES C9, hiAstrocytes C9 and HFA were seeded out in 96-well plates, in their respective media, at 60 K/cm^2^, 45 K/cm^2^ and 30 K/cm^2^, respectively. After 72 h cells were washed once with HBSS (w/ Ca^++^ and Mg^++^) and then incubated in either vehicle (DMSO) or inhibitors UCPH-101 (1.5uM, EAAT1 inhibitor) and WAY 213,613 (1uM, EAAT2 inhibitor) for 30 min in HBSS (w/ Ca^++^ and Mg^++^). After that, HBSS was changed with HBSS (w/ Ca^++^ and Mg^++^) containing 50uM of glutamic acid with either vehicle (DMSO) or inhibitors and incubated for 60 min; empty wells were also incubated with media containing 50uM of glutamic acid with either vehicle (DMSO) or inhibitors for glutamate uptake analysis. Samples were analyzed with the glutamate assay kit. Following sample collection, cells were incubated with Image-IT^TM^DEAD Green™. Consequently, cells were fixed with 4% PFA and stained with Hoechst (1:2000). Images were captured within 24-48 h with ImageXpress Micro 10x (Molecular Devices, CA, USA). Live dead count was assessed by the Live/Dead module.

### Inflammatory Assay

NES, hiAstrocytes and HFA were seeded out in 96-well plates at 60 K/cm^2^, 45 K/cm^2^ and 30 K/cm^2^, respectively. After 48 h, cells were washed once with DPBS and challenged with 50 ng/ml IL-1β in their respective media; this dose is within the range that is frequently used to challenge hiPSC-derived astrocytes [26, 28, 29, 36]. For the basal conditions, cells were washed with DPBS, and media was replenished. After 24 h, samples were collected and snap-frozen. Samples were analysed using the Mesoscale system (U-PLEX plate) according to kit instructions. The condition termed “IL-1β 24 h” for analysis of ICAM-1 also followed the same procedure. Cell numbers were determined following the same procedure as in the glutamate assay.

### Glutathione Assay

NES, hiAstrocytes and HFA were seeded out at 60 K/cm^2^, 45 K/cm^2^ and 30 K/cm^2^, respectively, in T12.5 flasks in their respective media for 48 h; cells were washed with DPBS all cells were changed to N2B27 with low EGF (1 ng/ml) and B27 without antioxidants, control media was also included in a cell-free T12.5 flask. After 24 h, media samples were collected from all cells and control media, and cells were harvested and snap-frozen. Media samples and cell suspensions were analyzed kinetically with the Glutathione assay kit (Sigma Aldrich, MO, USA) according to the manufacturer’s instructions with the following modifications: the kinetic reactions were analyzed for 30 min and the calibration curve was adjusted to include more points. The cell-free media flask values were subtracted from the media samples for extracellular quantification. Deproteinized samples were reconstituted in 0.5 M NaOH, and following complete reconstitution, samples were diluted to 100 mM NaOH, and total protein content was measured using the BCA kit.

The cell number/mg protein ratio was calculated separately by manually counting cells through a hemocytometer (each sample was counted using two independent samplings). For each sampling, four squares were counted. A known number of cells were lysed according to the Glutathione assay kit procedure described above. Deproteinized samples were reconstituted and analyzed as described above.

### Statistics

All statistical analyses were carried out in GraphPad Prism. Technical replicates refer to the same experimental procedure performed on different samples of the same differentiation round; biological replicates refer to the same experimental procedure repeated in “n” lines. Independent experiments refer to repeated independent differentiations, except for HFA samples, where different vials from the initial expansion were used.

## Results

### Neuroepithelial Stem Cells Generate Star-Shaped Astrocytes in 28 Days Under Astrocytogenic Conditions

NES are an intermediate cellular stage derived from induced pluripotent stem cells that generate neurons and glia. NES can be cryopreserved and cultured up to 100 passages without major phenotype changes. Hence, NES cells are a stable, robust starting point for neural differentiation [35].

ECM coating is pivotal and should be carefully considered for each target cell type. Stemming from the paradigm of how poly-L-ornithine specifically promotes neuronal differentiation [41], we wanted to test our hypothesis that a different coating and seeding density is required to encompass an astrocytic-boosting milieu for stable NES.

Our preliminary screening revealed that the conditions of TCW et al. 2017 [31] (Matrigel coating and 15 K/cm^2^ seeding density) induced astrocytic markers, to some extent, when applied to stable NES (Supplementary Fig. 1a-c). However, other mature (S100B and ALDH1L1) and functional astrocytic markers (SLC1A3) were not enriched (Supplementary Fig. 1d-f). When we replaced Matrigel with gelatin, it led to astrocytic enrichment of all tested astrocytic markers (Supplementary Fig. 1a-f). Gelatin, a denatured form of collagen, has been used for the primary isolation of astrocytes [42]; thus, we elected to use it in our differentiation strategy. 

The astrocytic mRNA levels aligned with cellular morphology; TCW et al. conditions (coating and seeding density) generated a largely fibroblastic morphology (Supplementary Fig. 2a), while gelatin at cell seeding density 15 K/cm^2^ generated astrocytes with some processes (Supplementary Fig. 2b). Gelatin at a cell seeding density 30 K/cm^2^ generated a higher proportion of star-shaped cells (Supplementary Fig. 2c).

Most interestingly, when we combined higher seeding density (30 K/cm^2^) on gelatin, astrocytic markers were highly enriched compared to previously tested conditions (TCW et al. Matrigel coating and 15 K/cm^2^ seeding density, [31]), suggesting that the differentiation media is indeed only a piece of a puzzle (Supplementary Fig. 1a-f, Gelatin 30 K/cm2 vs. TCW et al. conditions). Hence, gelatin and appropriate cell-to-cell contact seem to boost astrocytic traits synergistically. Furthermore, when we applied our optimized protocol on early NES (p.#13), astrocytic markers were considerably lower compared to when applied on NES at a later passage (p.#24) (Supplementary Fig. 2d). Finally, a recent study also corroborates our findings, Alisch et al. documented that fetal neural stem cells increase their gliogenic potential by dampening neurogenic genes during successive cell division [43]. Hence, NES increase their gliogenic potential at higher passages, similarly to what has been shown for fetal neural stem cells.

We used three iPS-derived NES lines, NES C9, NES C7 and NES AF22, to further test our protocol. Two of these lines (NES C9 and NES AF22) have been previously used for astrocyte differentiation [36, 37]. Here, we differentiated NES for 28 days (Fig. 1a) using a primary astrocytic media (AM) that has been previously shown to induce astrocytic traits in various research groups [31, 32]. In this approach, the hormone (hydrocortisone and progesterone) and growth factor (bFGF and IGF-1) cocktail is tailored for astrocytic growth and maintenance [44–46].

On day 0, cells appeared to form small aggregated or loosely adhering cells (Fig. 1b, day 0); the day after, cells were firmly attached to the culture vessels, and cells were passaged (at 30 K/cm^2^) on day 6. The first 6 days are crucial for a successful astrocyte differentiation; during that time, cells assume a colony-like morphology, growing outwards. By day 7, cells lost their NES-like behavior; namely, cells did not grow in NES colonies upon passaging, appeared more elongated, and a small percentage of cells assumed a triangular morphology (Fig. 1b, day 7). By day 14, the frequency of triangular-shaped cells increased (Fig. 1b, day 14) while the growth rate decreased (compared to NES), suggesting a switch to differentiation over self-replication. By day 21, cellular processes could be detected emanating from the somata, resembling a star-shaped morphology (Fig. 1b, day 21). The proliferation rate was further reduced by day 26 (Fig. 1b, day 26), and cells transitioned to more noticeable morphological changes assuming a star-like morphology (Fig. 1b, day 27), apparent in all lines (Fig. 1c, (i) hiAstrocytes C9 (ii) hiAstrocytes C7 and (iii) hiAstrocytes AF22).

### HiAstrocytes have a Distinct Astrocytic mRNA Expression Profile that Differs from Spontaneously Differentiated Cells

**Fig. 2:**
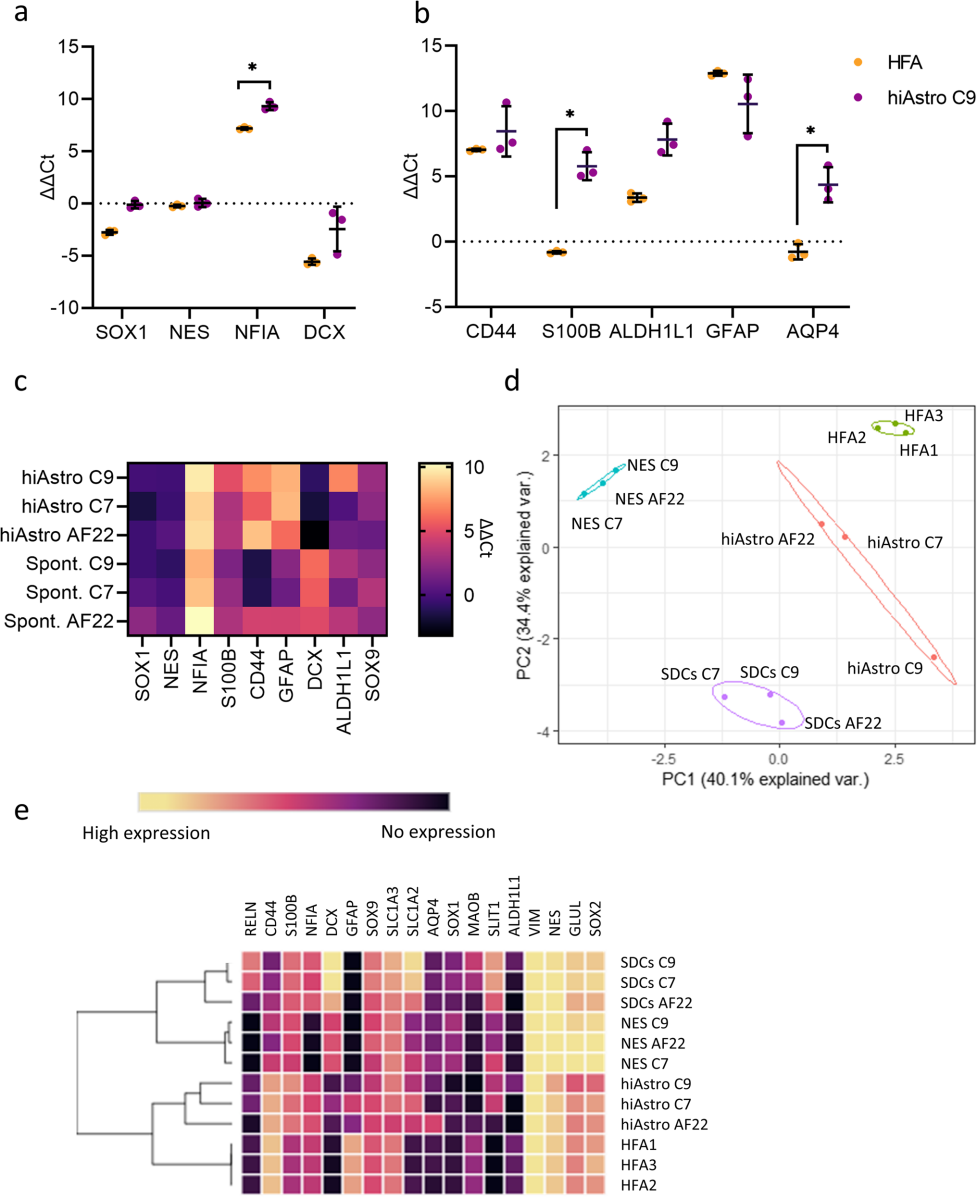
HiAstrocytes have a distinct astrocytic mRNA expression profile that differs from spontaneously differentiated cells. mRNA analysis (ΔΔCt values, vs. NES C9, normalized to GAPDH) of a) *SOX1*, *NES*, *NFIA* and *DCX* and b) astrocyte-specific markers *CD44*, *S100B*, *ALDH1L1*, *GFAP* and *AQP4* for hiAstrocytes C9 (purple) and HFA (orange). Data shown from n = 3 independent experiments. Error bars represent ± SD. Statistical analysis was done on the ΔCt values by using multiple unpaired student’s test (Holm-Šídák method) with Welch correction. *p < 0.05. c) Heatmap mRNA levels (ΔΔCt, vs. each respective NES line) of hiAstrocytes and SDCs from NES C9, C7 and AF22 d) PCA plot and e) Hierarchical clustering of NES C9, C7 and AF22, their corresponding differentiated astrocytes (hiAstro), spontaneously differentiated cells (SDCs) and HFA, color scale in ΔCt, GOI-house-keeping gene, highest expression, ΔCt = -1,17 for *VIMENTIN* in hiAstro C9, and no expression, ΔCt = 15.8 for *RELN* in NES C9). HFA from one isolation were used. Normalized ΔCt values were used for the PCA plot and hierarchical clustering, all Ct values above 35 were assigned to 35 to avoid overestimation of the results

As a first characterization of the hiAstrocytes, we used RT-qPCR and compared them to human fetal cortical astrocytes (HFA) at low passage (p.3). In hiAstrocytes C9, the so-called gliogenic switch, Nuclear factor IA (*NFIA*)[47], was upregulated during the differentiation while the neuronal progenitor marker *DCX* was downregulated (Fig. 2a), denoting glial commitment. Most astrocytic markers such as *CD44* and *ALDH1L1* were on a par with the expression levels in HFA, while *AQP4* and *S100B* were significantly enriched in hiAstrocytes, in contrast to HFA (Fig. 2b). Interestingly, NES and HFA had the same expression level of the astrocytic marker *S100B* (Fig. 2b). HiAstrocytes C7 and AF22 had an expression pattern comparable to hiAstrocytes C9 (Fig. 2c).

The canonical astrocytic marker *GFAP* was highly upregulated in hiAstrocytes and was expressed at similar levels as in HFA. Interestingly, changes in GFAP expression are associated with inflammatory conditions [48–50]*.* Hence, GFAP expression is crucial for accurate modeling in vitro.

Next, we sought to unveil the transcriptomic differences between the hiAstrocytes, and cells generated by spontaneous differentiation of the NES lines. Spontaneously differentiated cells (SDCs) were generated by growth factor withdrawal for 28 days on PLO-L2020 (Supplementary Fig. 3a). We elected to use PLO-L2020 for two reasons; gelatin does not support NES proliferation or spontaneous differentiation since neural stem cells do not adhere well to gelatin [51]. Secondly, we wanted to evaluate the confounding effects of media and coating on astrocytic markers.

*NFIA* was, upregulated in all cell lines in both hiAstrocytes and SDCs, while *CD44* expression was restricted to the hiAstrocytes (all lines) and SDCs AF22 (Fig. 2c). As expected, *DCX* was upregulated in all lines in SDCs; conversely, *DCX* was downregulated in hiAstrocytes (all lines). *GFAP* was highly upregulated in hiAstrocytes (all lines), while *GFAP* expression varied in SDCs. SDCs in AF22 showed higher *GFAP* expression than SDCs C9 or C7. The differential expression of *GFAP* and *CD44* in SDCs suggests a mixture of neurons and glia in SDCs AF22, while there is a neuronal enrichment in SDCs C9 and C7. In other studies, spontaneously differentiated NES AF22 has generated 86% neuronal cells[40] while NES C7 generated 76.4–90% neuronal cells[40, 52].

PCA of 18 genes analyzed by RT-qPCR showed a distinct separation between SDCs and hiAstrocytes, denoting the impact of the differentiation strategy on enriching astrocytic fate (Fig. 2d). HiAstrocytes clustered closer to HFA than NES or SDCs. Specifically, hiAstrocytes C7 and AF22 clustered closer to HFA than hiAstrocytes C9, while NES and SDCs had distinct clusters further away from HFA. Hierarchical clustering revealed that hiAstrocytes from all three lines clustered with HFA and were distinct from SDCs (Fig. 2e). We further elaborate on the line mRNA, protein, and functional differences in the discussion.

We also assessed the regionality of NES and hiAstrocytes. Gene expression analysis of two regionality markers, *RELN* and *SLIT1* (spinal cord markers, [53])*,* revealed that, strikingly, the NES lines have a different dorsoventral identity (Supplementary Fig. 3b). While all lines had no or insignificant expression of *RELN*, NES C7 seems to have a distinct regional identity denoted by the *SLIT1* expression, which remained almost unchanged during astrocyte differentiation, denoting a regionally patterned precursor that gives rise to specifically VA3 astrocytes. In hiAstrocytes C9, *RELN* and *SLIT1* were upregulated, while in hiAstrocytes AF22, there was a very low expression (*RELN* Ct = 33, *SLIT* Ct = 32). HFA also had a similar *RELN* expression pattern (Ct = 32), while *SLIT1* was not detected.

### Quantification of Astrocytic Markers and Processes Shows a Superior Protein and Phenotypic Profile Compared to HFA

**Fig. 3:**
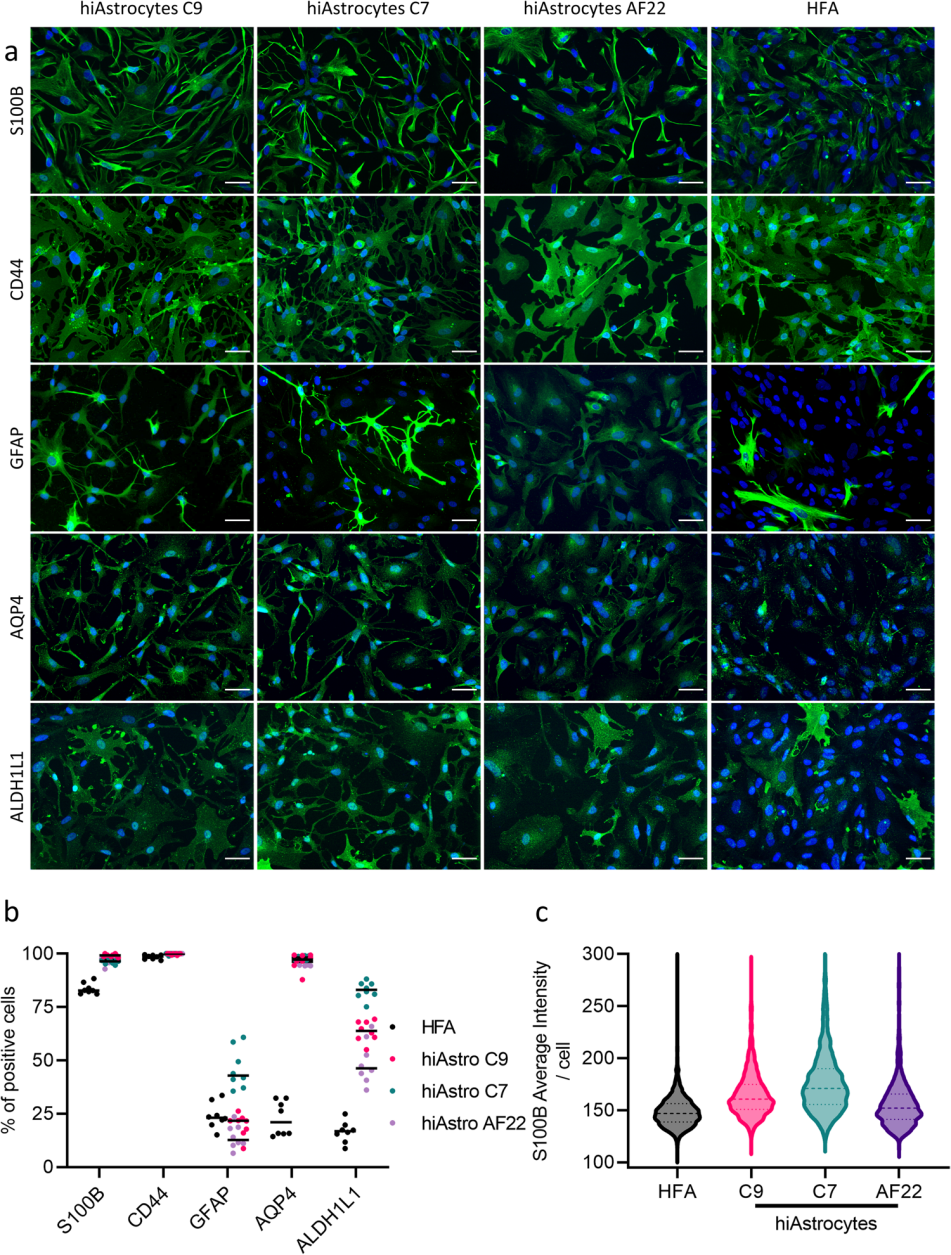
Quantification of astrocytic markers shows a superior protein profile compared to HFA. a) ICC images of astrocytic markers S100B, CD44, GFAP, AQP4 and ALDH1L1 for hiAstrocytes C9, C7 and AF22 and HFA. Scale bar 50 μm. b) Quantification of astrocytic markers S100B, CD44, GFAP, AQP4 and ALDH1L1; each dot represents one field of view. c) Violin plot depicting the average intensity (per cell) of S100B for hiAstrocytes. [C9 (1161 cells analyzed), hiAstrocytes C7 (1725 cells analyzed), hiAstrocytes AF22 (1295 cells analyzed) and HFA (2614 cells analyzed)]

To further assess the hiAstrocyte cellular phenotype, we performed immunocytochemistry and stained hiAstrocytes C9, C7, AF22 and HFA for the common astrocytic markers S100B, CD44, GFAP, AQP4, ALDH1L1 and the cytoskeletal marker VIMENTIN. All hiAstrocytes lines and HFA stained positive for CD44 (Fig. 3a), and all models reached almost 100% CD44^+^ cells (Fig. 3b). HiAstrocytes C9, C7, AF22 and HFA stained negatively for the neuronal marker DCX (Supplementary Fig. 3c).

HFA were 24% GFAP^+^ while hiAstrocytes C9, C7 and AF22 were 20%, 46% and 14% GFAP^+^, respectively (Fig. 3b). Quantification of ALDH1L1 revealed a low percentage (16%) of positive cells in HFA, while the quantification among the three lines was consistently higher than this, with hiAstrocytes C7 having the highest percentage of positive cells (83%) followed by hiAstrocytes C9 (64%) and AF22 (49%). HiAstrocytes from all three lines were positive for AQP4 with almost 100% positive cells (hiAstrocytes C9/C7/AF22, 96%/98%/96%), while HFA had a lower percentage of AQP4-positive cells (16%).

While hiAstrocytes and HFA were positive for S100B^+^, hiAstrocytes had a higher percentage of S100B^+^ astrocytes (hiAstrocytes C9/C7/AF22 vs. HFA, 99%/96%/97% vs. 84%). Intensity analysis of each population showed that hiAstrocytes C9 and C7 populations had higher average intensity per cell than HFA, as denoted by the shift in the violin plots (Fig. 3c). NES C9, NES C7 and NES AF22 stained positive for VIMENTIN only (Supplementary Fig. 4).

**Fig. 4:**
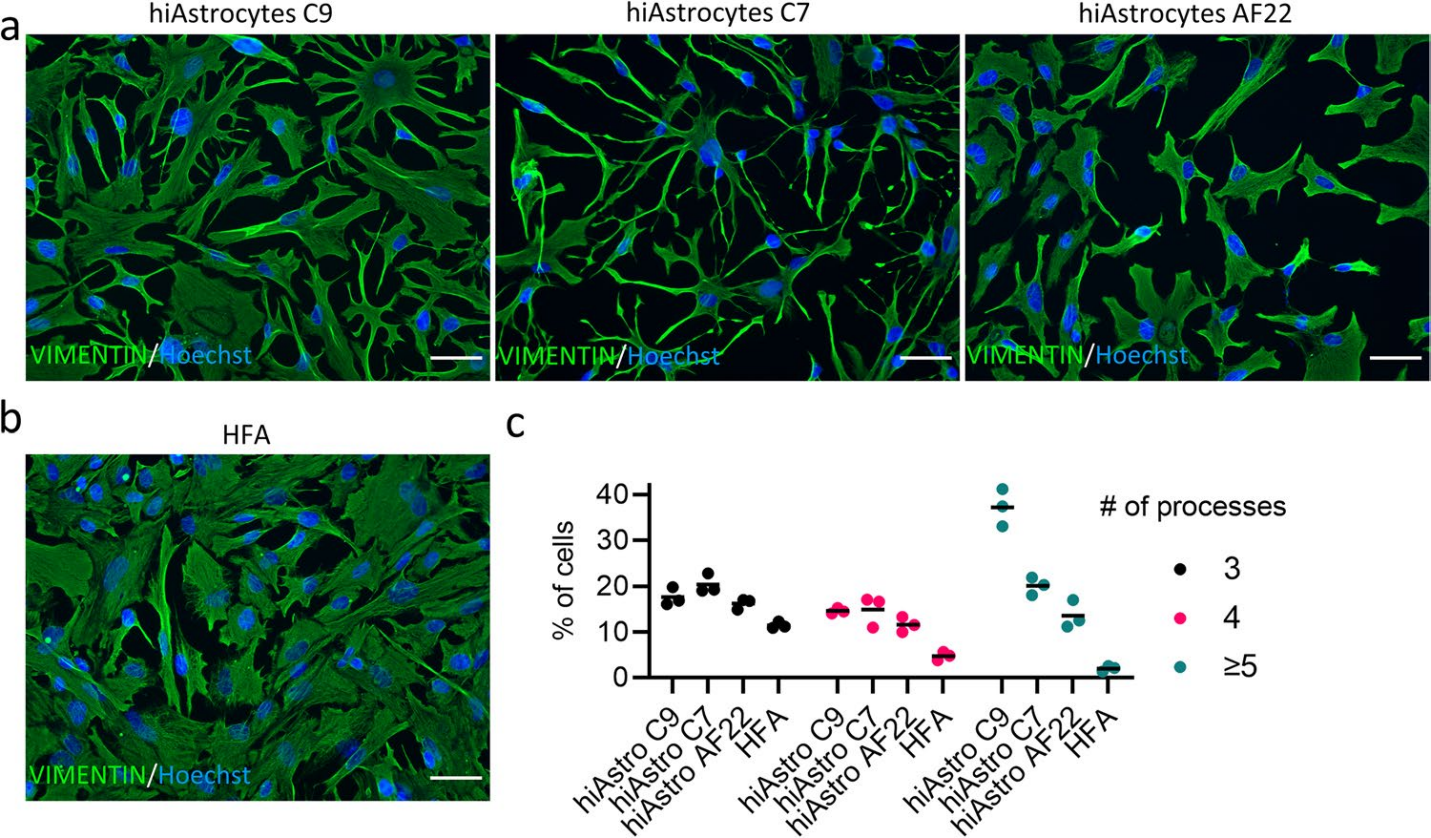
Quantification of astrocytic processes shows a more elaborate, star-shaped phenotype. ICC images of cytoskeletal marker VIMENTIN for a) hiAstrocytes C9, C7 and AF22 and b) HFA. Scale bar 50 μm. c) Quantification of astrocytic processes for hiAstrocytes C9, C7 and AF22, and HFA. Each dot represents the average of four fields of view (10x)

We further assessed the morphology of hiAstrocytes; a star-shaped morphology is likely to be important for in ﻿vitr﻿o disease modelling. For example, astrocytes remodel their processes and orient them towards lesions formed after head trauma or hemorrhage [14, 17]. Additionally, in transgenic AD animals, astrocytes exhibit reduced complexity in their processes [16] and in epilepsy, astrocytic processes appear thicker and longer [15]. We stained hiAstrocytes (C9, C7 and AF22, Fig. 4a) and HFA (Fig. 4b) with VIMENTIN to quantify astrocytic processes. HiAstrocytes had a higher percentage of cells with elaborate processes than HFA; specifically, HiAstrocytes C9 had the highest percentage of cells with multiple processes (69%, >  = 3, supplementary Fig. 5a). HiAstrocytes C7 and AF22 hovered around 55% and 41%, respectively, while HFA had only 18% cells with multiple processes.

On the higher end of the number of processes (> = 5), hiAstrocytes C9 showed the highest percentage (37%) of multiple processes (> = 5, Fig. 4c), followed by hiAstrocytes C7 (20%) and AF22 (13%) (> = 5, Fig. 4c). In contrast, only 2% of HFA exhibited multiple processes (> = 5, Fig. 4c).

### HiAstrocytes Exhibit Functional EAAT1- and EAAT2-Mediated Glutamate Uptake

**Fig. 5:**
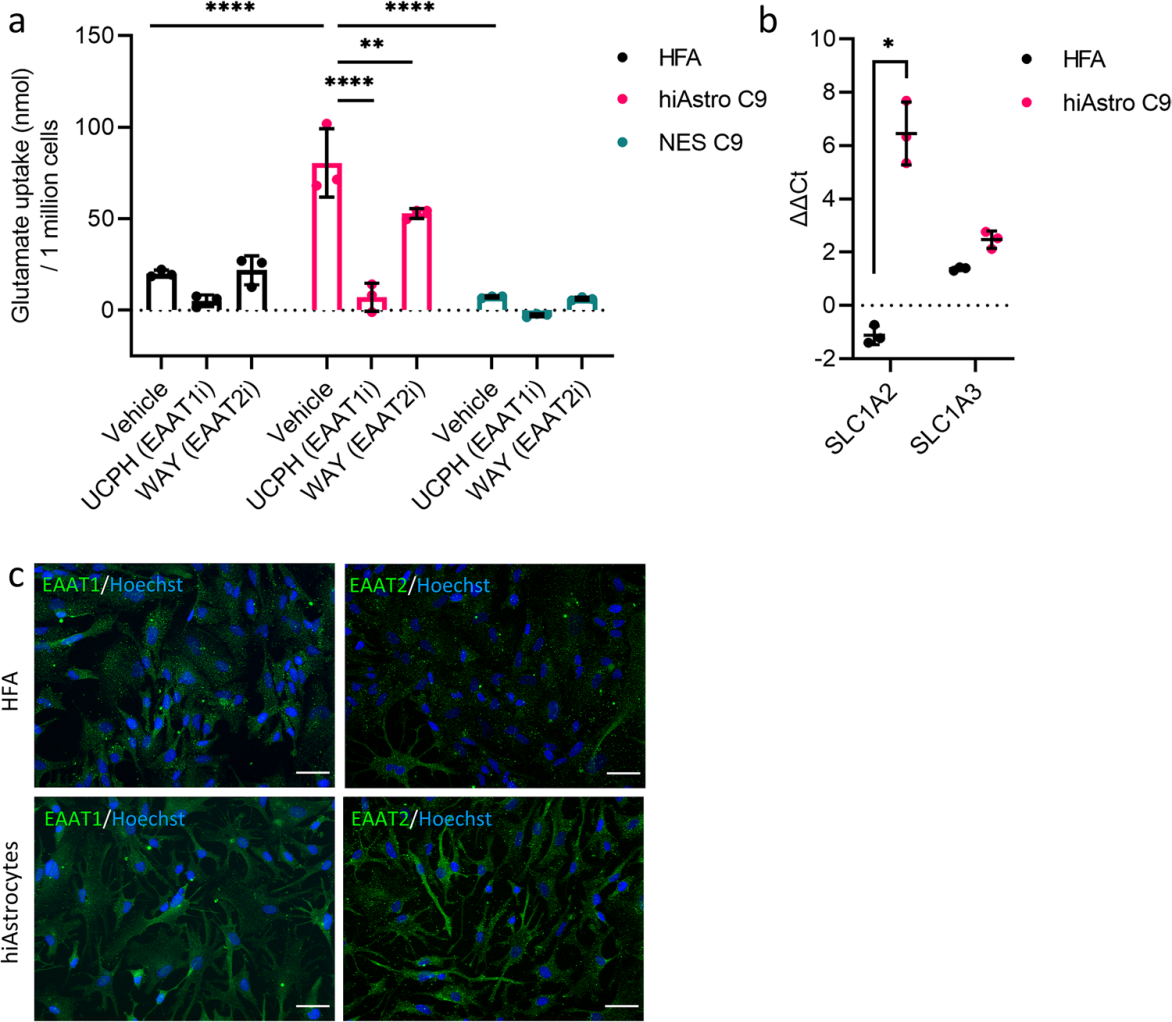
HiAstrocytes exhibit functional EAAT1- and EAAT2-mediated glutamate uptake. a) Glutamate uptake assay for HFA (black), hiAstrocytes C9 (pink) and NES C9 (green), n = 3 technical replicates. Error bars represent ± SD. Statistical analysis was done using two-way ANOVA followed by Tukey’s post-hoc test. *p < 0.05, **p < 0.01, ***p < 0.001, ****p < 0.0001. b) mRNA analysis of glutamate transporters *SLC1A2* and *SLC1A3* for hiAstrocytes C9 and HFA, n = 3 independent experiments. Error bars represent ± SD. Statistical analysis was done on the ΔCt values by using multiple unpaired student’s test (Holm-Šídák method) with Welch correction. *p < 0.05. c) ICC images of glutamate transporters EAAT1 and EAAT2 for hiAstrocytes C9 and HFA

Glutamate clearance is a fundamental process of astrocytes and is disturbed in pathological conditions. For example, in ALS, the glutamate transporter EAAT2 is lost in astrocytes suggesting a causal relationship between neuronal excitotoxicity and EAAT2 loss in astrocytes [13]; additionally, loss of functionality of glutamate transporters is associated with various other neurological disorders [54–56]. Hence, we next sought to characterize the glutamate uptake capacity of hiAstrocytes, a critical in vivo functionality of astrocytes. NES C9 showed 7.24 nmol glutamate uptake per million cells, while hiAstrocytes C9 showed more than a tenfold increase in glutamate uptake than NES (80.51 nmol per million cells, p < 0.0001, Fig. 5a). HiAstrocytes C9 showed four times higher glutamate uptake rate than HFA (20.03 nmol per million cells, p < 0.0001, Fig. 5a).

We used the non-substrate compounds UCPH-101 (UCPH) and WAY213613 (WAY) to inhibit the two astrocytic glutamate transporters EAAT1 and EAAT2, respectively. Both inhibitors significantly blocked glutamate uptake in hiAstrocytes compared to vehicle (vehicle vs. UCPH, 80.51 vs. 7.08 nmol per million cells, p < 0.0001 and vehicle vs. WAY, 80.51 vs. 52.89 nmol per million cells, p = 0.0057, Fig. 5a). In HFA, even though EAAT1 inhibition (UCPH) lowered glutamate uptake compared to vehicle (vehicle vs. UCPH, 20.00 vs. 5.14 nmol per million cells), it was not significant (p = 0.3117). HFA did not exhibit functional EAAT2 uptake with the WAY213613 inhibitor.

Further characterization of those transporters through RT-qPCR and ICC revealed that in hiAstrocytes C9 SLC1A2, the gene encoding for the EAAT2 transporter was highly upregulated (Fig. 5b) compared to HFA, which showed a slight downregulation (hiAstrocytes C9 vs. HFA, ΔΔCt 6.46 vs. -1.12). The transporter SLC1A3 had almost the same expression pattern in both cell populations. ICC of the two glutamate transporters showed that both hiAstrocytes and HFA are EAAT1^+^. HiAstrocytes stained positive for EAAT2 while HFA were weakly stained (Fig. 5c).

HiAstrocytes C7 and AF22 had glutamate uptake on par with HFA (hiAstrocytes C7/ hiAstrocytes AF22/ HFA, 16.12/ 17.33/ 20.00 nmol per million cells, supplementary Fig. 5b). Inhibition with UCPH decreased the uptake in hiAstrocytes C7; however, it did not reach significance (vehicle vs. UCPH, 16.12 vs. 8.68 nmol per million cells, p = 0.9585). HiAstrocytes C7 did not show EAAT2-mediated uptake (vehicle vs. WAY, 16.12 vs. 20.02 nmol per million cells. HiAstrocytes AF22 did not show any EAAT1-mediated uptake of glutamate (vehicle vs. UCPH, 17.33 vs. 21.61 nmol per million cells. Even though hiAstrocytes AF22 showed a reduced uptake when inhibited with WAY, it did not reach significance (vehicle vs. WAY, 17.33 vs. 11.49, p = 0.9903, Supplementary Fig. 5b).

### HiAstrocytes Harbor Unique Inflammatory Potency and Antioxidant Properties

A vital aspect of a differentiated cell’s repertoire is its capacity to respond to inflammatory agents, which should be on par with the in vivo counterpart, in our case, HFA. That is an important aspect of modeling brain pathologies in vitro. Therefore, we elected to challenge hiAstrocytes and HFA with IL-1b [57] and measured the secretion of Interleukin 6 (IL-6) and Interleukin 8 (IL-8) [58].

**Fig. 6:**
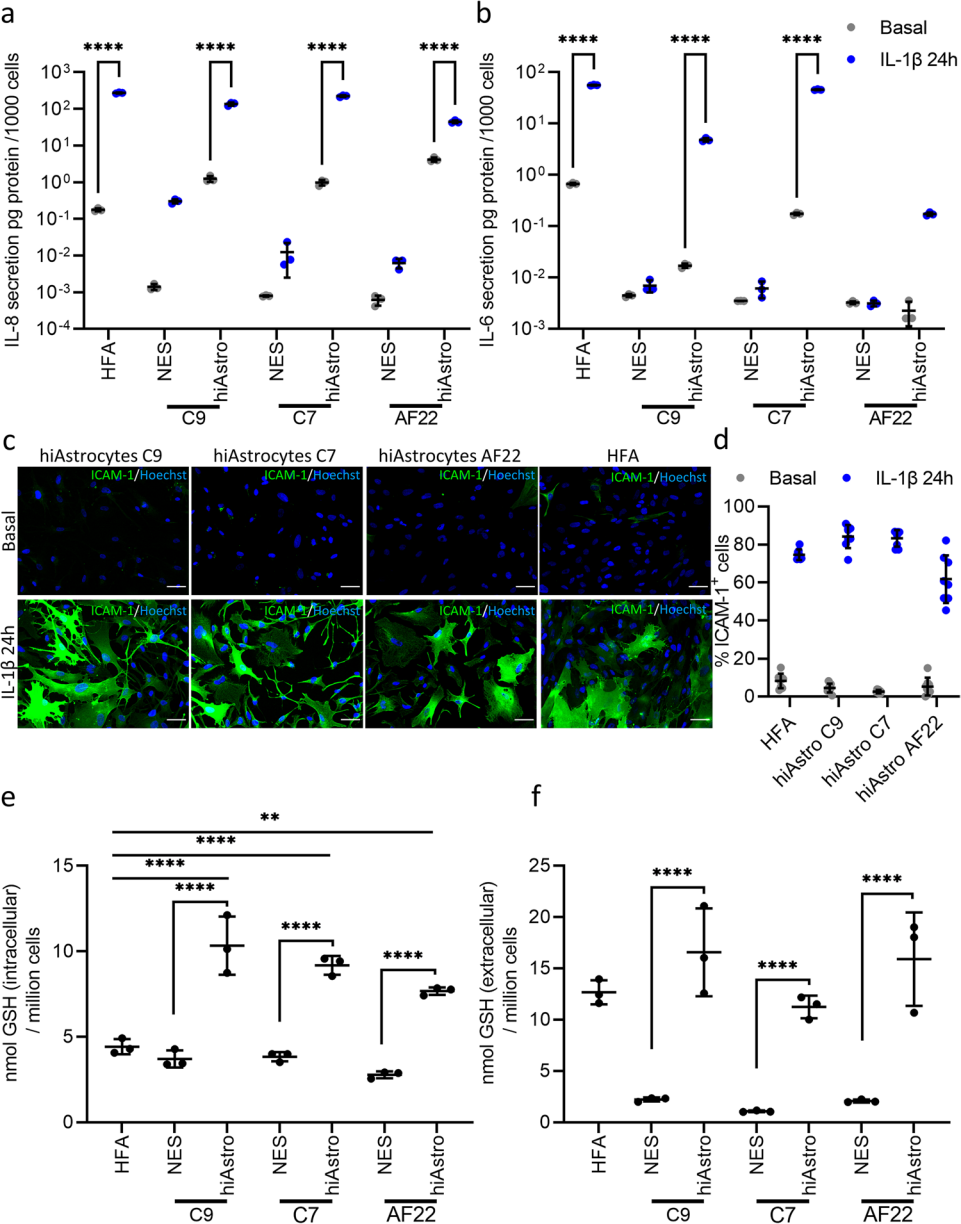
HiAstrocytes harbor unique inflammatory potency and antioxidant properties. Secretion levels of a) IL-8 and b) IL-6 in NES C9, C7 and AF22, their corresponding astrocyte differentiation (hiAstrocytes C9, C7 and AF22) and HFA under basal (grey) and inflammatory conditions (IL-1β 50 ng/ml for 24 h, blue), n = 3 technical replicates. c) ICC images of ICAM-1 for hiAstrocytes C9, C7 and AF22, and HFA under basal and inflammatory conditions (IL-1β 50 ng/ml for 24 h). Scale bar 50 μm. d) Quantification of ICAM-1^+^ cells under basal (grey) condition or under inflammatory stimuli (IL-1β 50 ng/ml for 24 h, blue). Glutathione levels e) intracellularly and f) extracellularly in NES C9, C7 and AF22, their corresponding astrocyte differentiation (hiAstrocytes C9, C7 and AF22), and HFA, n = 3 technical replicates. Error bars represent ± SD. Statistical analysis was done using two-way ANOVA followed by Tukey’s post-hoc test. *p < 0.05, **p < 0.01, ***p < 0.001, ****p < 0.0001

We first quantified the cytokine secretion of hiAstrocytes, quantification of IL-8 revealed that hiAstrocytes (all lines) and HFA responded to IL-1β by secreting IL-8 at a significant level compared to their basal conditions (p < 0.0001, Fig. 6a). IL-6 quantification showed that HFA and hiAstrocytes C7 had the highest secretion (56 pg and 47 pg per 1000 cells, respectively, Fig. 6b). HiAstrocytes C9 secreted 8 pg per 1000 cells, while hiAstrocytes AF22 secreted 0.171 pg per 1000 cells. IL-1β-challenged HFA, hiAstrocytes C9 and C7 had significantly higher IL-6 secretion compared to their basal conditions (p < 0.0001), while IL-6 secretion in hiAstrocytes AF22 challenged with IL-1β was not significantly higher compared to their basal conditions (Basal: 0.013 vs. IL-1β 0.171 pg per 1000 cells, Fig. 6b).

Apart from inflammatory cytokine secretion upon stimulation, e.g., astrocytes can also express ICAM-1 in inflammatory conditions [18] such as MS [59], brain injury and Alzheimer’s disease (AD) [60]. ICAM-1 expression under inflammatory conditions constitutes a mature immune response and exemplifies the immunomodulating capacity of astrocytes in the CNS. Upregulation and ICAM-1 ligation on astrocytes elicit a cascade of reactions, e.g., secretion of cytokines such as Tumor Necrosis Factor-alpha (TNFa) [61] and IL-6 [62]. Hence, we stained for ICAM-1 in basal and inflammatory conditions (IL-1β). ICC revealed that all lines and HFA showed weak staining of ICAM-1 in basal conditions, while when incubated with IL1-β for 24 h, the majority of cells were ICAM-1^+^ (Fig. 6c). Specifically, quantification of the ICC showed that in basal conditions, all hiAstrocytes and HFA had less than 10% ICAM-1^+^ (Fig. 6d). Upon inflammatory stimuli, hiAstrocytes C9 and C7 had the higher percentage of ICAM-1^+^ cells, 84% and 83%, respectively. HFA had slightly fewer positive cells, amounting to 75% ICAM-1^+^. HiAstrocytes AF22 had the lowest number of ICAM-1^+^, 61%.

Astrocytes synthesize and secrete GSH [63, 64], shielding CNS cells from oxidative stress, which is also reflected in the high astrocytic GSH content of ~ 8 mM [65]. Interestingly, GSH availability is altered in traumatic brain injury [19] and autism [20, 21], while GSH efflux is impaired in AD [22, 23]. A largely unexplored astrocytic aspect in stem cell-derived astrocytes is the capacity of astrocytes in vivo to protect the CNS against oxidative stress through GSH. Astrocytes have a very high GSH [65] that they continuously replenish since they constantly provide GSH precursors to neurons and brain endothelial cells [66].

We measured the total GSH content of NES, hiAstrocytes and HFA, and we also sampled the medium extracellularly to assess 1) the intracellular content of hiAstrocytes and how it compared to HFA GSH levels and 2) whether they can export it and thus extend their capacity to protect neighbouring cells against reactive oxygen species.

Our analysis showed that all hiAstrocytes showed significantly higher intracellular total GSH than the undifferentiated NES (p < 0.0001, Fig. 6e). When compared to HFA, hiAstrocytes had higher total GSH intracellularly, while HFA had 4.42 nmol total GSH per million cells, hiAstrocytes C9, C7 and AF22 showed 10.33 (p < 0.0001), 9.18 (p < 0.0001) and 7.68 nmol (p = 0.0014) total GSH per million cells, respectively.

Measurements of total extracellular GSH (Fig. 6f) revealed that all hiAstrocytes exported considerably more total GSH than their NES counterparts. While all NES lines hovered between 1.1–2.2 nmol total GSH per million cells, hiAstrocytes C9, C7 and AF22 showed a remarkable increase ranging from 11.2 to 16.6 nmol total GSH (p < 0.0001, Fig. 6f). The total extracellular GSH in hiAstrocytes C9 and AF22 was almost eight times higher than their respective NES lines. HiAstrocytes C7 showed an increase amounting to 10 times more than NES C7. Extracellular values of hiAstrocytes were on par with HFA.

## Discussion

Over the past years, there has been a plethora of astrocytic differentiation protocols reported, some more elaborate and time-consuming than others, spanning over many months or sorting steps [24, 28, 67]. Our differentiation strategy relies on creating an astrocytogenic milieu that brings forth astrocytic traits in only 28 days. The differentiation protocol we report is advantageous for CNS disease modeling, considering the simplicity and relatively cost-effective reagents and, most importantly, the cellular phenotype generated. We created the astrocytogenic milieu by combining an astrocytic-tuned ECM component, culture medium and cell-to-cell communication (regulated via seeding density) to generate functional and morphologically star-shaped astrocytes.

Collagen is an interesting ECM molecule; collagen is not abundant in the bulk ECM of the CNS [68] and is mainly restricted in the basement membrane and meninges. However, in vitro studies unveiled that astrocytes express and secrete fibrillar collagen-like proteins, which is inhibited in vivo by EGF signaling and meningeal cells [69, 70]. Interestingly, when fetal astrocytes were cultured in a 3D collagen gel, they assumed a star-like morphology instead of the elongated phenotype that is ubiquitous in conventional culturing environment [71]. This work shows how gelatin – a denatured formed of collagen – combined with a growth media designed to promote and sustain astrocytes creates an environment that can guide NES into functional star-shaped astrocytes with a mature phenotype. However, gelatin alone is too simplistic to fully recapitulate the very elaborate ECM during brain development. Nevertheless, our data suggest that gelatin, much like PLO for neurons[41], enhances astrocytic commitment in vitro.

Stable neural stem cells lines are crucial for the robustness and reproducibility of differentiations. Long-term neuroepithelial stem cells are stable neural precursors that can be cryopreserved and are a compelling starting point for neuronal and glial generation[35]. Conversely, unstable neural precursors usually ensue a limited life span and an unpredictable proliferation rate that limits their usefulness as a starting point to yield mature neurons or astrocytes.

Our previous work compared an astrocyte differentiation protocol and other astrocyte-like cells to human primary adult astrocytes per industry ethical policy [36]. However, fetal astrocytes are a more relevant model to compare human induced pluripotent stem cell (hiPSC)-derived astrocytes. Adult human astrocytes have undergone a developmental process that is very challenging to recapitulate in vitro; hence, astrocytic differentiations are most likely to generate astrocytes that have developmental proximity to human fetal astrocytes than to adult human astrocytes. Nonetheless, in vitro expansion of primary astrocytes follows an adaptation mechanism that may ensue a transcriptomic shift (i.e., dedifferentiation) similar to what has been seen with other CNS primary cells such as brain endothelial cells [72]. Hence, leveraging the potential of iPS technology, we created an astrocytic model that outperforms the model with the highest-to-date in vivo relevance, the HFA.

Our differentiation strategy generates astrocytes that resemble HFA both on an mRNA and protein level. HiAstrocytes and HFA are not in the same cluster in the PCA plot, albeit hierarchical clustering reveals that hiAstrocytes cluster with HFA and not with SDCs. We postulate that the reasons behind that deviation are: 1) the different developmental stages, 2) different regionality, and 3) stress and alternations of the cellular phenotype associated with the acute isolation of primary cells [73].

One clear example of the difference between hiAstrocytes and HFA is the expression of *S100B.* The S100B enrichment in hiAstrocytes corroborates our ICC data, where hiAstrocytes have a higher percentage of S100B^+^ cells than HFA. Interestingly, hiAstrocytes had a higher average intensity of S100B^+^ cells (per cell) than HFA. That could denote a developmental stage that surpasses the developmental stage of commercially available HFA (18–20 GW). In mice, *S100B* expression of GFAP^+^ astrocytes is associated with a mature state that lacks neural stem cell traits [74]. In humans, the transition from fetal to mature astrocytes is characterized by S100B upregulation [5]; moreover, the higher percentage of AQP4^+^ and ALDH1L1^+^ in hiAstrocytes reinforces the notion that hiAstrocytes have a developmental stage that transcends the 20 GW developmental stage of HFA [5].

Secondly, a largely unaddressed issue in most hiPSC-derived astrocyte protocols is the regionality of astrocytes. When modeling CNS diseases that affect specific brain regions, such as ALS, the proper regionality of astrocytes is a crucial component in attaining in vivo relevance. The importance of regionality in CNS diseases modeling is also exemplified by the region-dependent glutamate transporters' expression[75]. Transcriptomic analysis of two regionality markers associated with the spinal cord, *RELN* and *SLIT1* [53, 76, 77], revealed that the hiAstrocytes have a distinct regional identity; specifically, hiAstrocytes C9 appeared to be either a mixture of VA1, VA2 astrocytes or VA3. Interestingly, NES C7 was the only NES line that showed expression of *SLIT1,* which persisted during the differentiation associating hiAstrocytes C7 with ventrally located VA3 population. AF22 hiAstrocytes did not show expression of either *RELN* or *SLIT1*, suggesting a more anterior regional identity. HFA did not express any of these markers since these astrocytes were isolated from the cortex. However, more regionality markers should be explored to pinpoint the regionality of hiAstrocytes accurately. To what extent neural patterning affects the anteroposterior and dorsoventral identity of hiAstrocytes remains to be seen. Bradly et al. [78] showed that the regionality of neural stem cells influences the gene expression of downstream differentiation to astrocytes; hence, astrocytes derived from differentially patterned NES lines cannot possibly cluster very tightly owing to differential expression patterns in various regions of the brain.

The non-overlapping clusters in the PCA echo the differences mentioned above between hiAstrocytes and HFA; perhaps the inclusion of primary astrocytes of different parts of the brain would better associate with hiAstrocytes. Albeit having a different regional identity, all hiAstrocytes stained positive for the canonical astrocytic markers, S100B, AQP4, ALDH1L1 and CD44. Interestingly, the generated cells are GFAP^+^ which was not observed in our previous protocol to generate astrocytes from long-term proliferating NES [36, 37, 40]. NES (all lines) did not stain for any of these markers but were positive for VIMENTIN (Supplementary Fig. 4).

A noticeable difference between the NES lines in SDCs was the different populations they generated. NES C9 and C7 were biased towards neuronal commitment, while NES AF22 generated a mixture of glial and neuronal populations. Our data for NES corroborates other studies’ findings; specifically, NES AF22 produced a mixture of glial and neuronal populations upon growth factor withdrawal (86% neuronal vs. 14% glia) [40]. Additionally, spontaneous differentiation of NES C7 has been show to produce varying degrees of neuronal population (76.4–90% neuronal) [40, 52]. According to our results, NES AF22 should have a higher capacity for astrocyte differentiation than NES C7. However, NES AF22 underperformed compared to NES C9 and NES C7. Similar to what we report here, NES AF22 has previously underperformed compared to other lines when differentiated towards an astrocytic lineage [36]. We postulate that these differences could be attributed either to the different neural inductions used to generate the lines (NES C9 and C7: a variant of dual-SMAD and NES AF22: spontaneous neural induction) or to the type of reprogramming of the corresponding hiPSC lines (iPS C9 [79] and C7 [80] non-integrating Sendai virus and hiPSC AF22 integrating lentivirus). The former could be a plausible reason since differences in neural inductions have been shown to affect astrocytic potential [66].

The morphology of astrocytes is another salient feature of this cell type, mirroring their physiological/pathological state. A noteworthy pathological condition is schizophrenia, where researchers have shown that astrocytes in schizophrenia have a lower number of astrocytic processes than healthy astrocytes [81]. A star-shaped morphology has been greatly elusive in shorter differentiation protocols [31, 36] and is only possible to attain after prolonged differentiation ~ 5 months, as Oksanen al. [25] documented or when transplanting hiPSC-derived astrocytes in mice [82]. HiAstrocytes showed a star-like morphology and had more complex morphologies (processes >  = 3, 41—69%, line depended, Supplementary Fig. 5a) than HFA (18%), making this differentiation strategy ideal for CNS disease modeling in vitro where astrocytic processes are affected by pathological conditions [14–16].

EAAT1 expression and functionality has been documented previously in NES-derived astrocytes [36]. HiAstrocytes C9 showed specific EAAT1 glutamate uptake and had a similar expression pattern to HFA. EAAT2 is not expressed in HFA in vitro [83], and its expression is induced by co-culturing primary astrocytes with neurons [84, 85] or brain endothelial cells [86]. Interestingly, our results show that EAAT2 was significantly upregulated in hiAstrocytes C9 compared to HFA, without using molecular inducers such as Ceftriaxone [83] or co-culturing with other CNS cells. The glutamate assay and the specific EAAT2 inhibitor, WAY213613, revealed that hiAstrocytes C9 had functional EAAT2-mediated transporters. We postulate that the differentiation strategy, more specifically, the high density that cells are kept before each passage and the presence of bFGF in the differentiation media, work synergistically toward EAAT2 expression and functionality. Notch signaling has been shown to increase GLT-1 expression in mice [86], and the same has been shown for bFGF [87]. Other groups have shown EAAT2 expression in iPS-derived astrocytes [27, 67, 88], and here we report on an astrocytic model that shows specific EAAT2 expression and functionality. EAAT2 has gained attention since studies suggest that excitotoxicity-induced neuronal death is closely correlated with neurological disorders [54] such as ALS and AD [13, 55, 56]. In autism, the significance of EAAT2 over EAAT1 has been documented in a mouse model [89]. Thus, EAAT2 expression and functionality are pivotal when attempting to model in vitro these neurological disorders. Ceftriaxone is one candidate that showed promising results in vitro and in animal models in increasing GLT-1 expression [90, 91]. Even though this specific compound failed in clinical trials, compounds that modulate EAAT2 expression are still a plausible therapeutic route for ALS [92]. The other two lines performed similarly to HFA. NES AF22 has been shown to have limited capacity for astrocytic potential [36, 93]. On the mRNA level, SLC1A2 and SLC1A3 in hiAstrocytes C9 were highly upregulated compared to hiAstrocytes C7 and AF22 (Supplementary Fig. 5c). Specifically, for SLC1A2, hiAstrocytes C9 showed 40-fold upregulation from the NES stage, while hiAstrocytes C7 and hiAstrocytes AF22 showed an 8-fold and 15-fold upregulation, respectively. HiAstrocytes C9 was the only line that showed an upregulation of SLC1A3 (8-fold upregulation). The expression levels of SLC1A3 remained generally stable during the differentiation, with a 1.5-fold increase in hiAstrocytes C7 and no change in hiAstrocytes AF22.

HiAstrocytes C7, hiAstrocytes AF22 and HFA had similar expression patterns, translating into a glutamate uptake in the same order of magnitude (16–20 nmol per million cells, Supplementary Fig. 5b). The expression level differences of glutamate transporters between the lines are also reflected in the amount of glutamate uptake. In this study, a 60 min assay was developed; a longer assay could be needed for lines that do not upregulate glutamate transporters significantly; hence, glutamate assays should be developed after considering the expression levels of glutamate transporters in each line.

Regardless, caution should be taken when choosing lines for downstream glutamate uptake assays. The type of neural induction and reprogramming of the parental lines could interfere with potential downstream functionality in glutamate clearance. Expression levels of glutamate transporters can give a lead in cellular functionality.

HiAstrocytes exhibited strong inflammatory potency. Upon IL-1b simulation, hiAstrocytes secreted IL-6 and IL-8. Other reports have also shown IL-6 and IL-8 secretion for hiPSC-derived astrocytes [24, 26, 28]. In our study, we quantitatively analyzed and compared cytokine secretion between hiPSC-derived astrocytes and primary fetal astrocytes. This differentiation study is unique because our results showed that hiAstrocytes C9 and C7 secreted IL-8 and IL-6 at comparable levels to HFA. Hence, this differentiation strategy is well-suited for studies of astrocytic involvement during neuroinflammation since hiAstrocytes performed similarly to HFA.

Importantly, upon IL-1b stimulation, hiAstrocytes expressed ICAM-1, suggesting that hiAstrocytes have a mature inflammatory phenotype. Additionally, NES (all lines) were not responsive when challenged with IL-1β staining negative for ICAM-1 (Supplementary Fig. 6a). ICAM-1 expression has never been shown before in a hiPSC-derived astrocytic model. ICAM-1 is expressed in pathological conditions such as MS [59], brain injury and AD[60]. Specifically, in MS, ICAM-1 on astrocytes directly communicates with infiltrating leukocytes [62, 94] and microglia [95]. ICAM-1 upregulation is also an important link in the relay of inflammatory stimuli between contact-mediated and secreted cytokines; activation of ICAM-1 has been shown to elicit TNFa and IL-6 secretion in astrocytes [61, 62].

The intracellular GSH content of hiAstrocytes and HFA is on par with studies on primary astrocytes (Supplementary Fig. 6b), ranging from 16 to 50 nmol/mg protein [96–98]. HiAstrocytes C9 and C7 hovered around the higher end of that range (57 nmol/mg protein), while hiAstrocytes AF22 and HFA had lower GSH content, 35 and 24 nmol/mg protein, respectively. Our differentiation approach generates astrocytes that exhibit superior export of GSH compared to other hiPSC-derived astrocytes. One other study that measured extracellular GSH in hiAstrocytes documented ~ 8.13 nmol/mg protein [25], while our study generated astrocytes that exported at least six times more GSH (Supplementary Fig. 6c and 6d). The rate of GSH export is hiAstrocytes ranged between 2.9 to 3.8 nmol GSH/ (h x mg protein) (Supplementary Fig. 6e). HFA showed an export rate of 2.9 nmol GSH / (h x mg protein).

Interestingly, our values are in the range of previous studies in rat primary astrocytes, 3.2 nmol / (h x mg protein) [99]. HiAstrocytes C9 and C7 exhibited ~ 7 times more GSH export than their respective NES lines, and hiAstrocytes AF22 ~ 10 times more GSH than NES AF22 (in nmol GSH / million cells). Astrocytes continuously synthesize GSH; the highest consumption of GSH occurs in the form of GSH export. Hence, astrocytes need to continuously synthesize GSH to compensate for exported GSH. The intracellular content of GSH is defined by 1) the rate that cells synthesize GSH from precursor molecules and 2) the rate that cells export GSH. Contemplating the similar export rates between hiAstrocytes and HFA and considering that all cells were cultured in the same media (i.e., same availability of GSH precursors), hiAstrocytes showed 2 to 3 times more GSH content in 24 h compared to HFA. This difference suggests that HFA lag and cannot fully compensate for the GSH export; hence, hiAstrocytes have a superior capacity to mitigate insults and reactive oxygen species.

The strategic location of astrocytes in the brain, covering over 90% of the brain's blood vessels with their processes, makes astrocytes the first line of defense against xenobiotics and toxins that enter the brain. Neurons cannot synthesize GSH independently and rely on astrocytes as a GSH source [100]. Moreover, astrocyte-secreted GSH counteracts the detrimental effects of an insult to the blood–brain barrier [66]. ROS are prevalent in many, if not all, neurological conditions [101–105]; are they part of the etiology or disease progression? Are astrocytes the cause or the domino factor in disease progression? GSH cycle disturbances can potentially shed light on this front.

Astrocytes harbor in their immune response a repertoire that transcends mere cytokine secretion; astrocytic responses are characterized by a cascade of reactions that involve morphological rearrangement of their processes and expression of adhesion molecules (e.g., ICAM-1), loss of glutamate transporters and GSH redox balance shift. Consequently, this hiPSC-derived astrocytic protocol constitutes a multifaceted in vitro model that may serve as a powerful tool enabling pathological cues to surface and potentially further deepen our knowledge of how astrocytes are involved in the etiology, onset, and progression of CNS pathological conditions.

## Conclusions

We report on specific astrocytic traits that have not been assembled before in a hiPSC-derived astrocyte generation. This conveniently short differentiation protocol based on comparably affordable reagents and a stable neural precursor may contribute to the generation of CNS disease models with improved biofidelity and the advancement of personalized medicine.

## Supplementary Information

Below is the link to the electronic supplementary material.

Supplementary Figure 1 Evaluation of astrocyte differentiation strategies using qPCR analysis. mRNA expression of astrocytic markers a) *GFAP* b) *CD44* c) *SOX9* d) *S100B* e) *ALDH1L1* and f) *SLC1A3* for differentiated NES C9 on Matrigel and 15K/cm^2^ (TCW et al. 2017 conditions, Matrigel and 15K/cm^2^ seeding density) and on gelatin in seeding densities 15 and 30K/cm^2^, all data retrieved after 28 days of parallel differentiations in the specified conditions. All conditions originated from the same NES culture. (n=1 independent experiment). Supplementary Figure 2 Comparison of the various differentiation strategies and passage impact on astrocytic commitment. Brightfield images after 28 days of differentiation of NES C7 in AM medium under conditions a) Matrigel and 15K/cm^2^ seeding density (TCW et al. conditions), gelatin with b) seeding density 15K/cm^2^, and c) 30K/cm^2^. Scale bar 100 μm. d) mRNA expression comparison of astrocytic markers between astrocyte differentiations from early passage (p.#13) and later passage NES (p.#24). ΔΔCt analysis was done using NES from the specific line at the corresponding passage (n=1 independent experiment). Supplementary Figure 3 Characterization of SDCs and hiAstrocytes a) Brightfield images after 28 days of spontaneous differentiation of i) NES C9 (SDCs C9) ii) NES C7 (SDCs C7) and iii) NES AF22 (SDCs AF22). Scale bar 100 μm. b) Relative expression (2^ΔCt^) of *SLIT1* (y-axis) and *RELN* (x-axis) for hiAstrocytes, NES lines and HFA c) DCX staining of hiAstrocytes C9, C7, AF22 and HFA. Scale bar 50 μm. Supplementary Figure 4 ICC of the NES lines used in this study. ICC images of NES lines NES C9, C7 and AF22 for the astrocytic markers S100B, CD44, GFAP, ALDH1L1. Scale bar 50 μm (20x). Supplementary Figure 5 Quantification of astrocytic processes and glutamate uptake a) Quantification of astrocytic processes of HiAstrocytes C9 (pink), C7 (green), AF22 (purple) and HFA (black), reuse of data from Fig. 4b to exemplify the more elaborate processes of hiAstrocytes vs. HFA. Each dot represents an average of four fields of view (10x). b) Glutamate uptake assay for HFA (black), hiAstrocytes C9 (pink), hiAstrocytes C7 (green) and hiAstrocytes AF22 (purple), n = 3 technical replicates. Error bars represent ±SD. HFA and hiAstrocytes data points were reused from Figure 5a for comparison with hiAstrocytes C7 and AF22. c) mRNA expression of glutamate transporters *SLC1A2* and *SLC1A3* for hiAstrocytes HFA (black), hiAstrocytes C9 (pink), hiAstrocytes (green) and hiAstrocytes AF22 (purple), n = 3 technical replicates. Error bars represent ±SD. ΔΔCt values for hiAstrocytes were determined by using the respective NES line as a reference, ΔΔCt values for HFA were determined by using the average ΔCts of the 3 NES lines. Supplementary Figure 6 Characterization of immune potency of NES and quantification of glutathione levels of hiAstrocytes and HFA. a) ICAM-1 staining for basal and inflammatory conditions for NES C9, C7 and AF22. Scale bar 50 μm. b) Total intracellular and c) total extracellular glutathione content for hiAstrocytes C9, C7, AF22 and HFA normalized by total protein content, n=3 technical replicates. Error bars represent ±SD. Statistical analysis was done using two-way ANOVA followed by Tukey’s post-hoc test. *p < 0.05, **p < 0.01, ***p < 0.001, ****p < 0.0001. d) Comparison with the work from Oksanen et al. (2017), n = 3 biological replicates, the dot in Oksanen study is the mean of the obtained values (n=14 biological replicates) in their study derived by dividing by the molecular weight of glutathione. The glutathione determination in Oksanen et al. (2017) was done according to Liddell et al. (2006) [106], while in this study, a glutathione assay kit was used (Sigma-Aldrich). e) Glutathione efflux rate of hiAstrocytes C9, C7, AF22 and HFA.(PDF 9254 KB)

## Data Availability

Data analyzed in the current study are available from the authors on reasonable request.

## Abbreviations

AD: Alzheimer’s disease
ALS: Amyotrophic lateral sclerosis
AM: Astrocyte media
CNS: Central Nervous System
DTI: Defined trypsin inhibitor
ECM: Extracellular matrix
GSH: Glutathione
HFA: Human fetal astrocytes
hiAstrocytes: Human iPS-derived Astrocytes
hiPSCs: Human induced pluripotent stem cells
ICAM-1: Intercellular adhesion molecule 1
IL-1β: Interleukin 1 beta
IL-6: Interleukin 6
IL-8: Interleukin 8
MS: Multiple sclerosis
NES: Neuroepithelial stem cells
PLO: Poly-L-ornithine
TNFa: Tumor Necrosis Factor alpha
