# Supplementary material for: Generation of Human iPSC-Derived Astrocytes with a mature star-shaped phenotype for CNS modeling
Source: Stem Cell Rev Rep. 2022 Apr 30;18(7):2494–512. doi: 10.1007/s12015-022-10376-2 (PMC9489586; doi:10.1007/s12015-022-10376-2)
Supplement: Supplementary file 1 — Supplementary Figure 1 Evaluation of astrocyte differentiation strategies using qPCR analysis. mRNA expression of astrocytic markers a) GFAP b) CD44 c) SOX9 d) S100B e) ALDH1L1 and f) SLC1A3 for differentiated NES C9 on Matrigel and 15K/cm2 (TCW et al. 2017 conditions, Matrigel and 15K/cm2 seeding density) and on gelatin in seeding densities 15 and 30K/cm2, all data retrieved after 28 days of parallel differentiations in the specified conditions. All conditions originated from the same NES culture. (n=1 independent experiment). Supplementary Figure 2 Comparison of the various differentiation strategies and passage impact on astrocytic commitment. Brightfield images after 28 days of differentiation of NES C7 in AM medium under conditions a) Matrigel and 15K/cm2 seeding density (TCW et al. conditions), gelatin with b) seeding density 15K/cm2, and c) 30K/cm2. Scale bar 100 μm. d) mRNA expression comparison of astrocytic markers between astrocyte differentiations from early passage (p.#13) and later passage NES (p.#24). ΔΔCt analysis was done using NES from the specific line at the corresponding passage (n=1 independent experiment). Supplementary Figure 3 Characterization of SDCs and hiAstrocytes a) Brightfield images after 28 days of spontaneous differentiation of i) NES C9 (SDCs C9) ii) NES C7 (SDCs C7) and iii) NES AF22 (SDCs AF22). Scale bar 100 μm. b) Relative expression (2ΔCt) of SLIT1 (y-axis) and RELN (x-axis) for hiAstrocytes, NES lines and HFA c) DCX staining of hiAstrocytes C9, C7, AF22 and HFA. Scale bar 50 μm. Supplementary Figure 4 ICC of the NES lines used in this study. ICC images of NES lines NES C9, C7 and AF22 for the astrocytic markers S100B, CD44, GFAP, ALDH1L1. Scale bar 50 μm (20x). Supplementary Figure 5 Quantification of astrocytic processes and glutamate uptake a) Quantification of astrocytic processes of HiAstrocytes C9 (pink), C7 (green), AF22 (purple) and HFA (black), reuse of data from Fig. 4b to exemplify the more elaborate processes of hi [file 12015_2022_10376_MOESM1_ESM.pdf]

Supplementary Figure 1

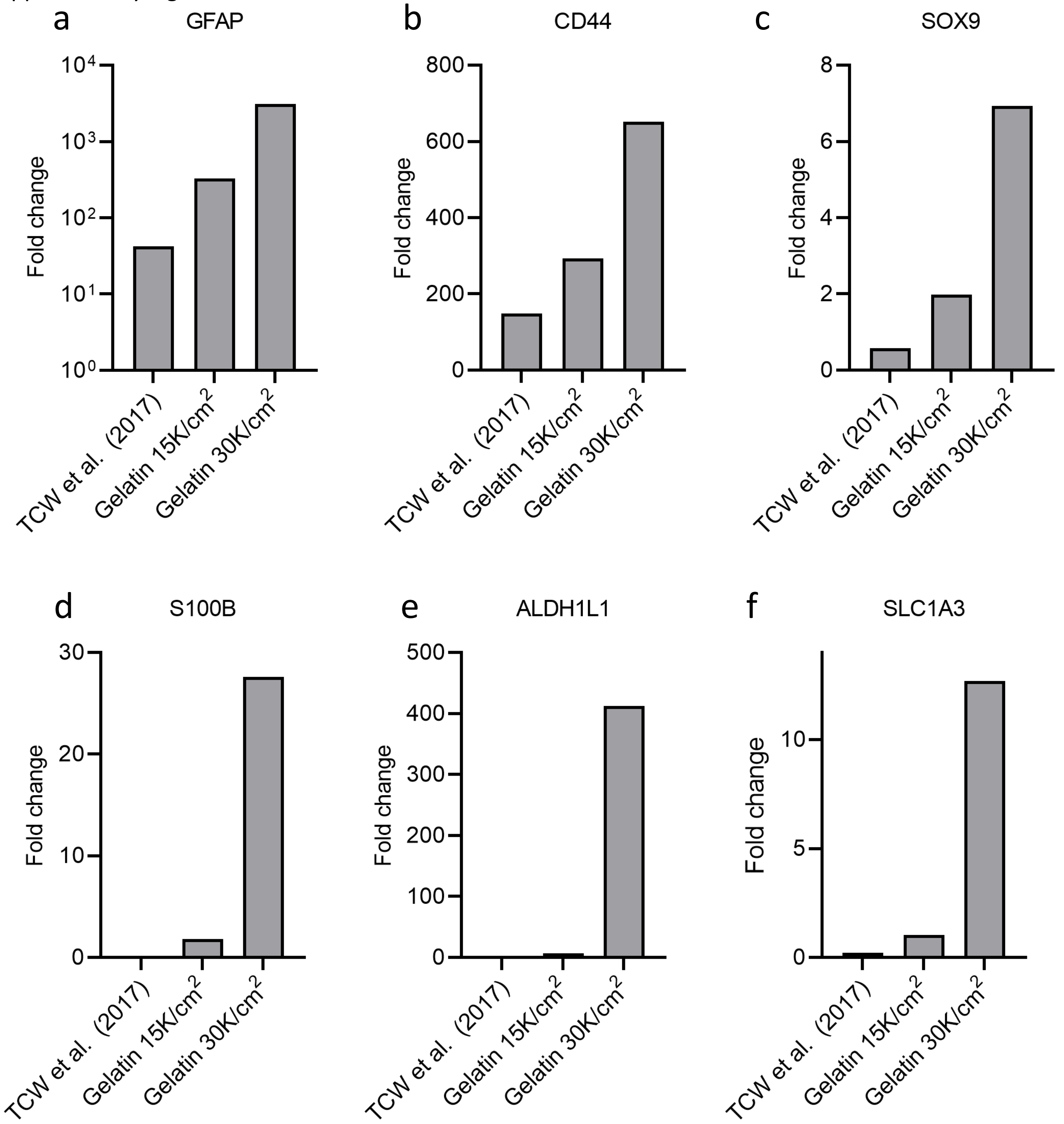

Supplementary Figure 2

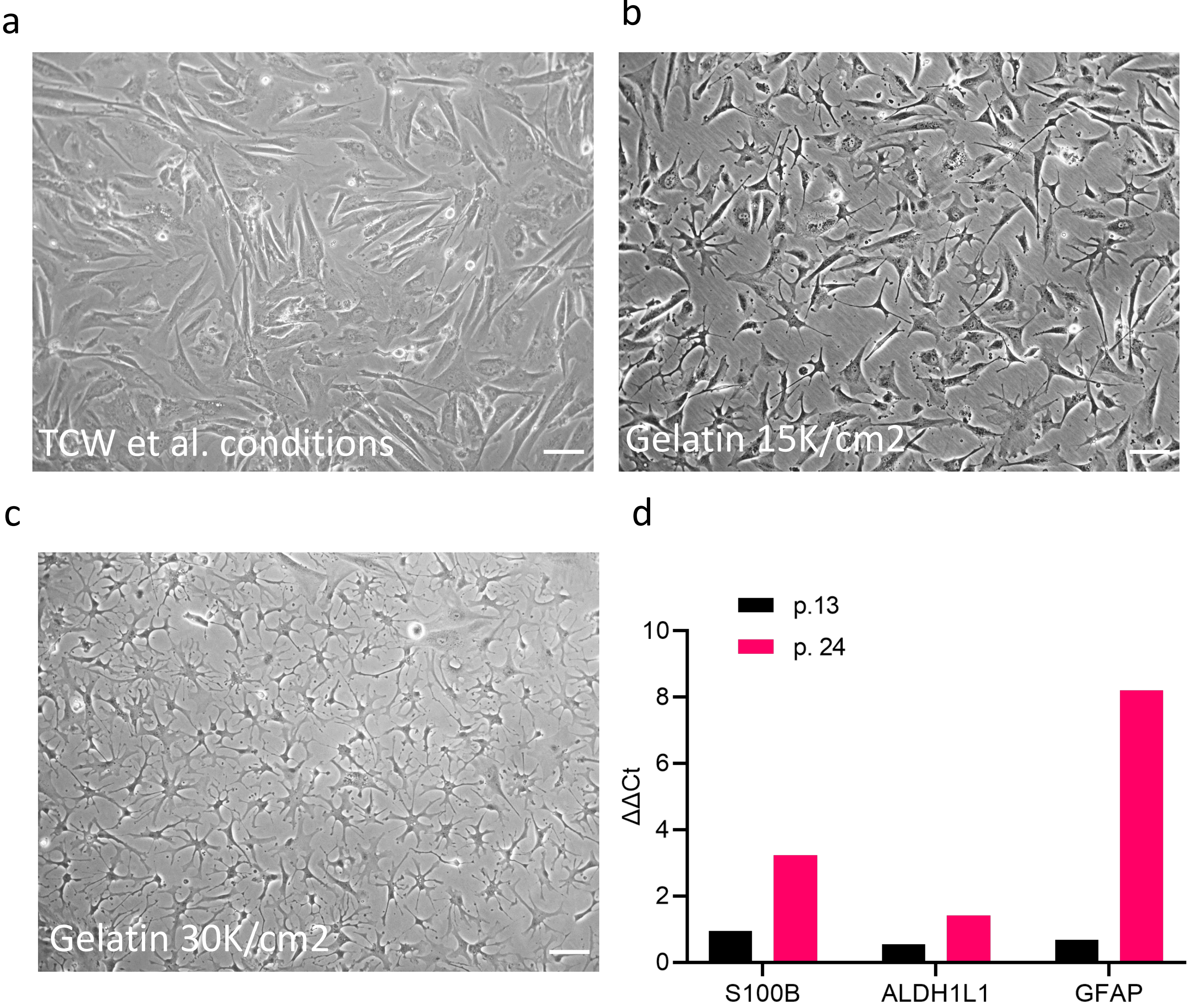

Supplementary Figure 3

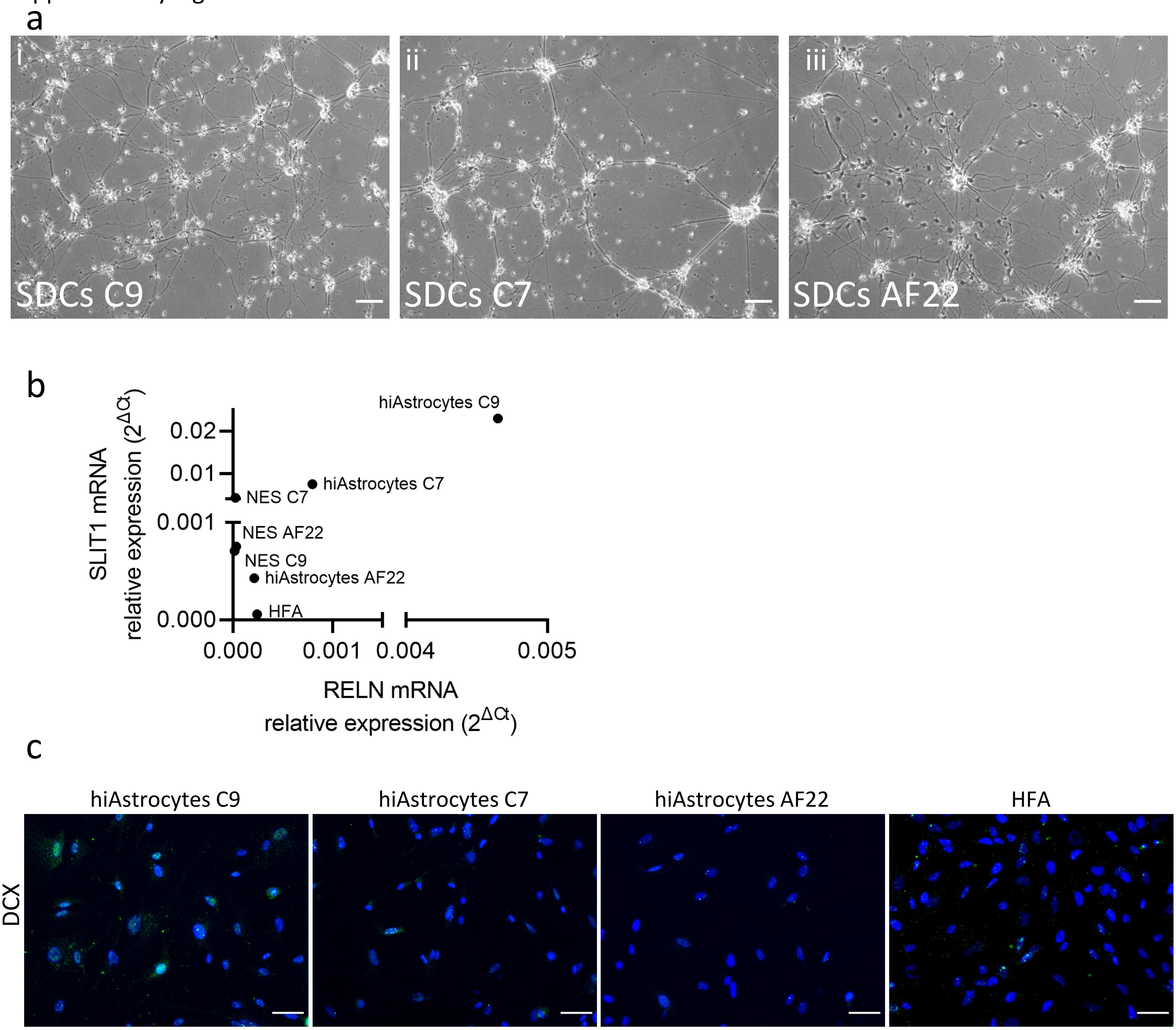

Supplementary Figure 4

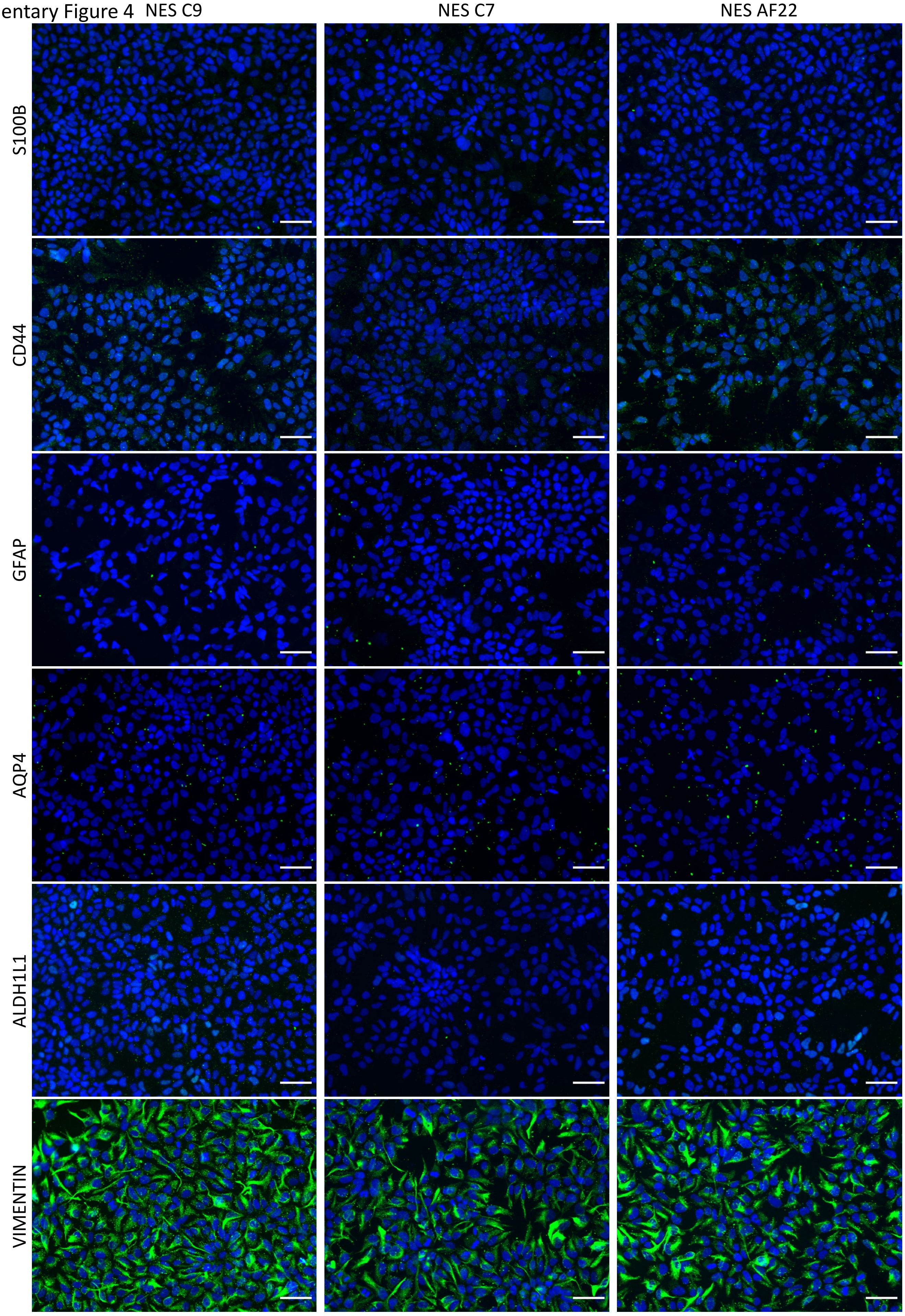

Supplementary Figure 5

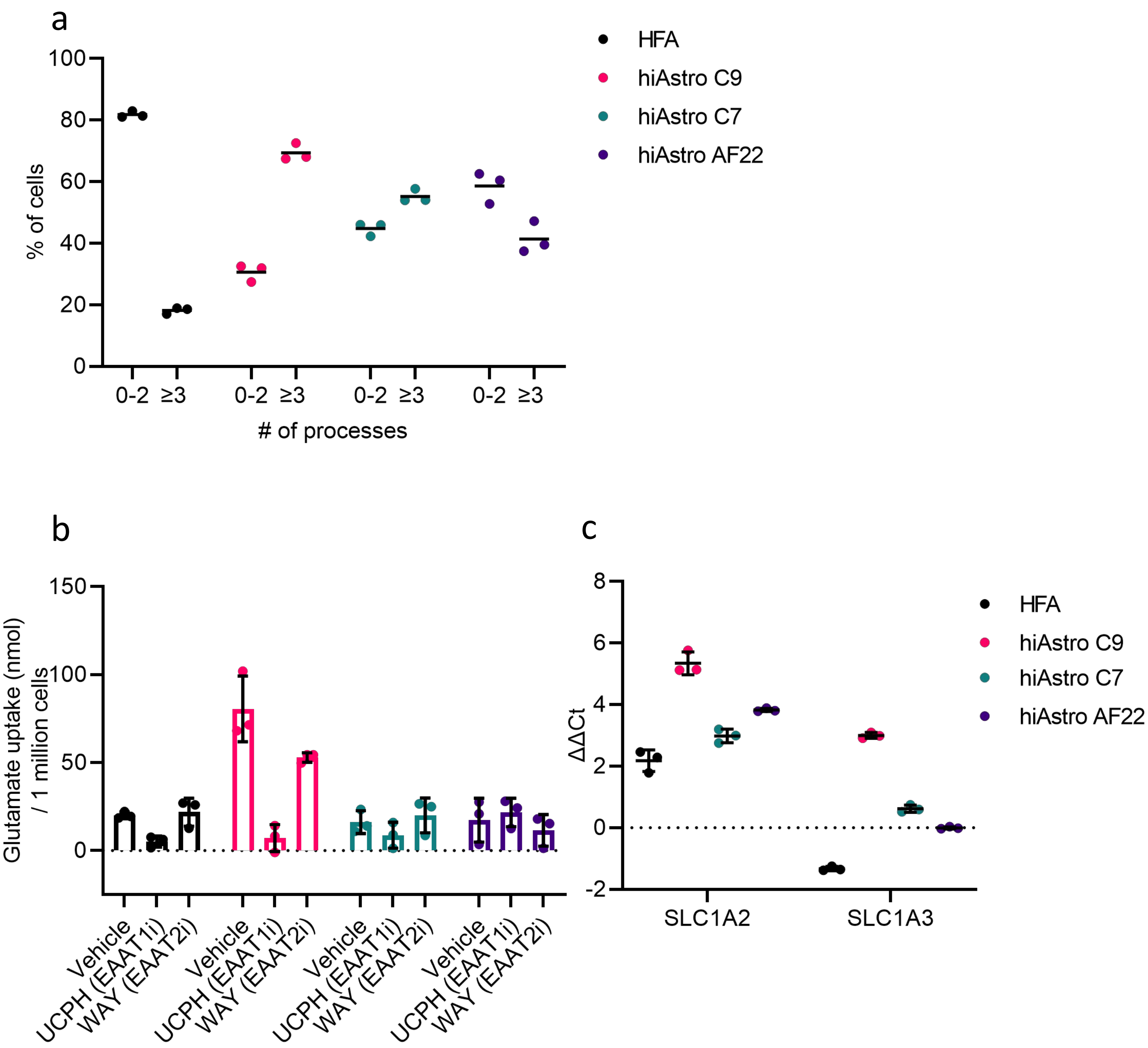

Supplementary Figure 6

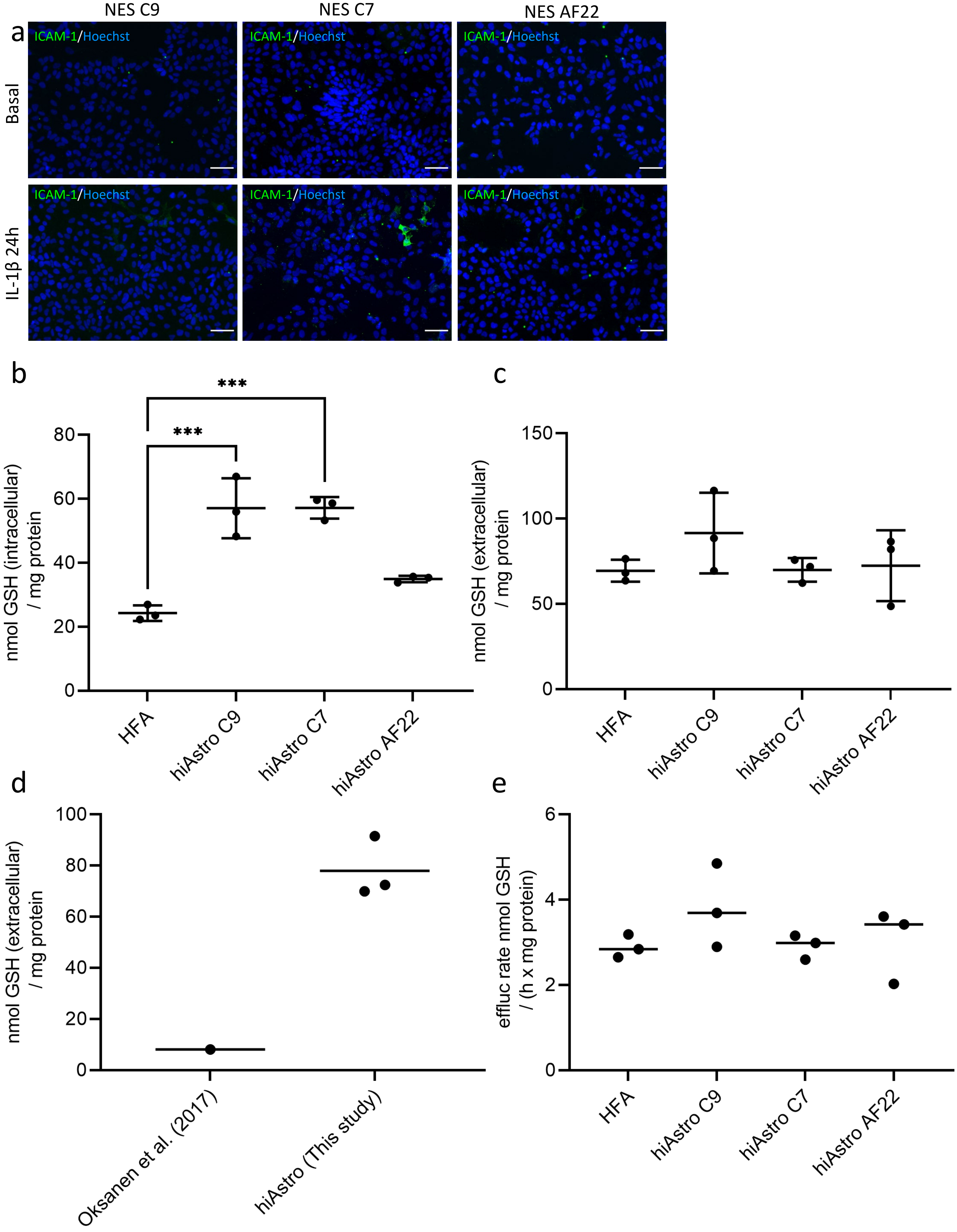

Supplementary Table 1 List of reagents and kits

Reagent	Supplier	Catalogue Number
DMEM:F12 Glutamax	Thermo Fisher	31331-028
N2	Thermo Fisher	17502-048
B27	Thermo Fisher	17504-044
B27, minus antioxidants	Thermo Fisher	10889-038
bFGF	R&D Systems	233-FB
EGF	Sigma-Aldrich	E9644
PLO	Sigma-Aldrich	P3655
L2020	Sigma-Aldrich	L2020
DPBS (w/o Ca^{++} and Mg^{++})	Thermo Fisher	14190-094
DTI	Thermo Fisher	R-007-100
AM kit	ScienCell	1801
DPBS (w/ Ca^{++} and Mg^{++})	Thermo Fisher	14040-091
Hoechst 33342	Thermo Fisher	H3570
Triton X-100 (1%)	Thermo Fisher	HFH10
Glutamate detection kit	Abcam	ab138883
Glutathione detection kit	Sigma-Aldrich	CS0260
HBSS	Thermo Fisher	14025-050
DMSO	Sigma-Aldrich	D2650
Glutamic acid	Sigma-Aldrich	49449
UCPH 101	Abcam	ab120309
WAY 213613	R&D Systems	2652
IL-1β	R&D Systems	201-LB
TrypLE Select	Thermo Fisher	12563-029
Gelatin	Thermo Fisher / ScienCell	S-006-100 / 0423
BDNF	R&D Systems	248-BDB
GDNF	R&D Systems	212-GD
Goat serum	Sigma-Aldrich	G9023
Poly-D-Lysine	Sigma-Aldrich	P6407
Goat anti-rabbit CF448	Sigma-Aldrich	SAB4600044
Goat anti-mouse CF555	Sigma-Aldrich	SAB4600302

Supplementary Table 2 List of cell lines used

Cell line	Gender	iPSC reprogramming	Age	Neural induction method	Publication
Control 9	Male	mRNA	Child	Dual SMADi + CHIR99021	Navarro et al. 2020
Control 7	Male	Sendai virus	Middle age	Dual SMADi + CHIR99021	Lam et al. 2019
Control AF22	Female	Lenti virus	Middle age	Spontaneous UM, colony picking	Falk et al. 2012

Supplementary Table 3 List of antibodies used in this study

Antibody	Supplier	Catalog	RRID	Dilution
S100B	Abcam	ab52642	AB_882426	1:100
CD44	Abcam	ab157107	AB_2847859	1:500
GFAP	DAKO	Z0334	AB_10013382	1:500
AQP4	Sigma-Aldrich	HPA014784	AB_1844967	1:125
ALDH1L1	Abcam	ab190298	AB_2857848	1:500
VIMENTIN	Abcam	ab92547	AB_10562134	1:500
EAAT1	Thermo Fisher	PA5-19709	AB_10982702	1:200
EAAT2	Thermo Fisher	711020	AB_2633106	1:250
ICAM-1	R&D Systems	BBA3	AB_356950	1:50

Supplementary Table 4 List of TaqMan probes used in this study.

Gene	TaqMan Assay ID
ALDH1L1	Hs01003842_m1
CD44	Hs01075864_m1
DCX	Hs00167057_m1
GAPDH	Hs02758991_g1
GFAP	Hs00909233_m1
NES	Hs04187831_g1
NFIA	Hs00325656_m1
S100B	Hs00902901_m1
SLC1A2	Hs01102423_m1
SLC1A3	Hs00904823_g1
SOX1	Hs01057642_s1
SOX2	Hs01053049_s1
RELN	Hs01022646_m1
SOX9	Hs00165814_m1
MAOB	Hs01106246_m1
SLIT1	Hs00171488_m1
VIM	Hs05024057_m1
GLUL	Hs01013055_g1
AQP4	Hs00242342_m1
